# Perturbed transcriptional profiles after chronic low dose rate radiation in mice

**DOI:** 10.1371/journal.pone.0256667

**Published:** 2021-08-24

**Authors:** Hildegunn Dahl, Dag M. Eide, Torstein Tengs, Nur Duale, Jorke H. Kamstra, Deborah H. Oughton, Ann-Karin Olsen

**Affiliations:** 1 Department of Infection Control and Environmental Health, Norwegian Institute of Public Health, Oslo, Norway; 2 Centre for Environmental Radiation (CERAD), Norwegian University of Life Sciences (NMBU), Ås, Norway; 3 Faculty of Veterinary Medicine, Department of Population Health Sciences, Institute for Risk Assessment Sciences, Utrecht University, Utrecht, The Netherlands; University of South Carolina, UNITED STATES

## Abstract

Adverse health outcomes of ionizing radiation given chronically at low dose rates are highly debated, a controversy also relevant for other stressors. Increased knowledge is needed for a more comprehensive understanding of the damaging potential of ionizing radiation from all dose rates and doses. There is a lack of relevant low dose rate data that is partly ascribed to the rarity of exposure facilities allowing chronic low dose rate exposures. Using the FIGARO facility, we assessed early (one day post-radiation) and late (recovery time of 100–200 days) hepatic genome-wide transcriptional profiles in male mice of two strains (CBA/CaOlaHsd and C57BL/6NHsd) exposed chronically to a low dose rate (2.5 mGy/h; 1200h, LDR), a mid-dose rate (10 mGy/h; 300h, MDR) and acutely to a high dose rate (100 mGy/h; 30h, HDR) of gamma irradiation, given to an equivalent total dose of 3 Gy. Dose-rate and strain-specific transcriptional responses were identified. Differently modulated transcriptional responses across all dose rate exposure groups were evident by the representation of functional biological pathways. Evidence of changed epigenetic regulation (global DNA methylation) was not detected. A period of recovery markedly reduced the number of differentially expressed genes. Using enrichment analysis to identify the functional significance of the modulated genes, perturbed signaling pathways associated with both cancer and non-cancer effects were observed, such as lipid metabolism and inflammation. These pathways were seen after chronic low dose rate and were not restricted to the acute high dose rate exposure. The transcriptional response induced by chronic low dose rate ionizing radiation suggests contribution to conditions such as cardiovascular diseases. We contribute with novel genome wide transcriptional data highlighting dose-rate-specific radiation responses and emphasize the importance of considering both dose rate, duration of exposure, and variability in susceptibility when assessing risks from ionizing radiation.

## Introduction

Ionizing radiation (IR), both natural (i.e., radon, cosmic, soil, and food) and human-made (i.e., medical, nuclear industry, and power plant accidents), are recognized as hazardous for human health and the environment [1]. Some of the most important studies, building the basis for the biological understanding of radiation-induced health effects, involve high doses and high dose rates with a short duration of exposure. These studies include the long-term follow-up of the atomic (A)-bomb survivors (Life Span Study; LSS) [2–4]. The LSS is recognized as a reliable source of epidemiological data due to cohort size, exposures of both genders at all ages, and a wide spectrum of individually assessed doses, although given over a short time and at high dose rates.

The epidemiological evidence for increased cancer risks with radiation dose from studies of the A-bomb survivors [5–7] largely supported the Linear No-Threshold (LNT) risk assessment model [4]. This model assumes that cancer risks after low doses of ionizing radiation (<100 mGy) can be extrapolated linearly from acute high dose data as a no-threshold exponent. The use of this model in the low dose area is highly debated [8,9]. Accumulating evidence indicates that disease progression following low total dose or low dose rates (<6 mGyh^-1^) [10–14] may be different from high dose and high dose rate exposures.

Besides cancer, high levels of radiation exposure also lead to a range of non-cancer effects [15–17], like circulatory (cardiovascular and stroke) [18,19] and metabolic diseases. Concerning the liver, observations of the Hiroshima and Nagasaki A-bomb survivors showed increased incidences of both cancer and fatty livers [7,20]. Increased incidences of non-cancer effects are also identified after low levels of radiation [21–23]. Several of these effects emerge late after exposure, but inconsistencies and confounding factors make associations difficult [15].

A dose of radiation given over a short period is more effective in producing certain kinds of biological damage, such as double-strand breaks (DSB) than when the same dose is delivered over a more extended period [24–27]. Radiation brings about cellular damage directly, indirectly, and non-targeted [28,29]. The *direct* hit of the radioactive photon can lead to a broad spectrum of damage and alterations to both DNA and other cellular molecules. *Indirect effects* arise from hydrolysis, generating highly reactive oxygen species (ROS) capable of reacting with every molecule in the cell. ROS is a well-known radiation-induced mechanism of toxicity. If the antioxidant capacity is overwhelmed, cellular oxidative stress may result in oxidation of cellular components, initiating response cascades to restore cellular integrity. *Non-targeted effects* are seen in cells not directly exposed to the radiation and are characterized as genomic instability and bystander effects [30]. It is suggested that indirect and non-targeted effects may play a more critical role after exposure to low total doses and low dose rates [31] and that the cellular implications from the direct effects might be negligible. However, the significance of disease manifestation from radiation-induced indirect and non-targeted effects is still debated.

Radiation exposure activates and inhibits numerous transcriptional pathways in response to different exposure regimes (low or high dose; acute, chronic, or protracted exposure). Knowledge regarding the molecular events mediating responses is critical to understand radiation toxicity. There is great emphasis on genetic mutations and chromosomal aberrations following radiation-induced DNA damage as the main mechanism contributing to increased cancer incidence and genetic instability. However, modulators may exist that could change the levels of disease risk [32]. DNA methylation has been proposed as a modulator, affecting gene transcription via silencing gene expression directly. Evidence indicates that these mechanisms are widely involved in ionizing radiation response [33]. Various exposure regimes are shown to induce different patterns of gene expression after high and low dose and dose rates [34]. A comprehensive understanding of these radiation-induced molecular events is essential as the transcriptional response may play a role in health outcomes [32] and could act as biomarkers of exposure and response [35].

We expect hepatic signaling pathways to be modulated by high dose rate ionizing radiation at the transcriptional level and that this could be identified through genome-wide transcriptional profiling. We hypothesize that the transcriptional profile is modulated differently after chronic low dose rate ionizing radiation when given the same total dose as the high dose rate, thus impacting other biological signaling pathways. Our study is designed to address this hypothesis by a) investigating the hepatic transcriptional response following whole-body exposure to low and high dose rate ɣ-irradiation given to the same high total dose (3 Gy), b) identifying dose rate-specific perturbed functional pathways, c) detecting strain-specific dose and dose rate radiation responses; and d) evaluating the impact of dose rate on the DNA methylation status.

## Materials and methods

### Animals and housing

Specific Pathogen Free CBA/CaOlaHsd and C57BL/6NHsd male mice (called CBA and B6 throughout the article), purchased from Envigo (Horst, The Netherlands), were used (3–8 weeks old at arrival). The mice were acclimatized for a minimum of four days after delivery and randomly housed in groups of five. Mice from each line were mixed in the cages (2–3 per mouse strain). They were housed in individually ventilated disposable PET plastic cages (IVC racks) (Innovive, San Diego, USA) under controlled temperature and light conditions (21±2°C, 45±15% relative humidity, 50 air changes h^-1^ and 12h light phase) with *ad libitum* access to tap water in PET bottles and SDS RM1 feed (Special Diet Services, Essex, UK). Due to space limitations in the radiation field, the mid dose rate (MDR) groups were housed in disposable PET cages like the other groups but using transport lids outside the IVC rack during the radiation exposure. Aspen tree bedding (Nestpack, Datesand Ltd., Manchester, UK) was used in all cages. At termination, the mice were administered anesthesia using ZRF-cocktail (Zolazepam, Tiletamine, Xylazine, and Fentanyl) followed by heart puncture and collection of blood (EDTA coated S-Monovette®, Sarstedt, Germany) before cervical dislocation and collection of tissues. The tissues were snap-frozen in liquid nitrogen and stored at -80°C until use. Care of animals and experimental protocols were in adherence to the national legislation for animal experimentation and approved by the Norwegian Food Safety Authority (NFSA, Approval no. 8803). No mice died or showed clinical signs due to the exposure.

### Experimental design

**Fig 1:**
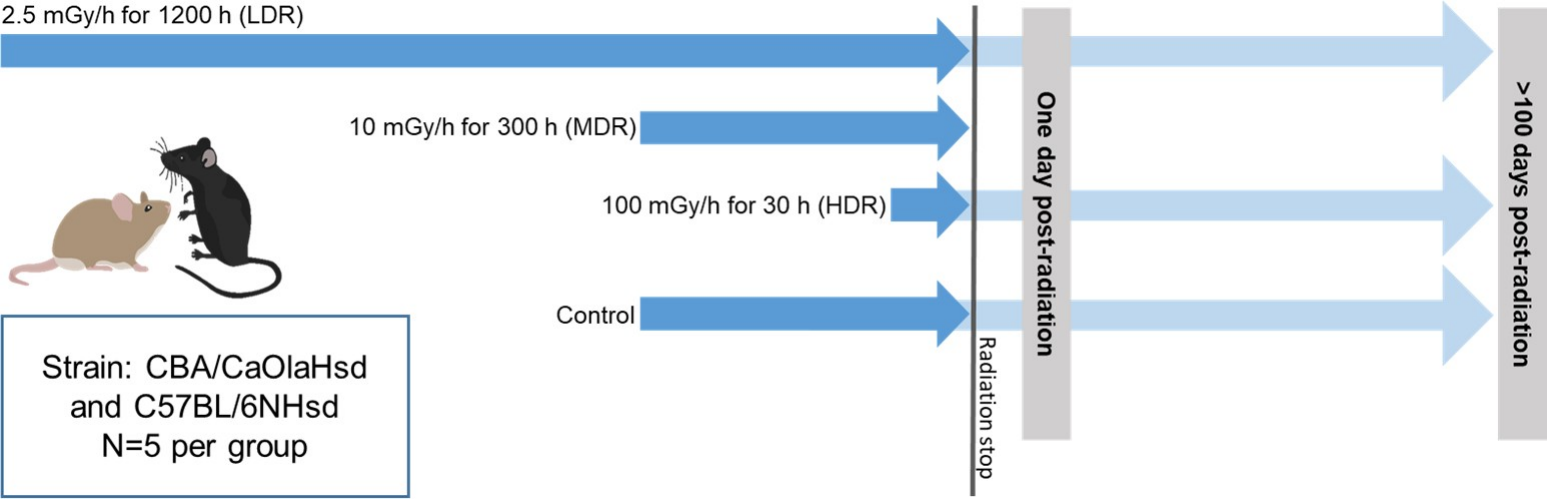
Schematic overview of the experimental study design. The study includes studying the effects of ɣ-radiation (^60^Co) at two time points after exposure: One day post-radiation and >100 days post-radiation (Late effect). Gamma radiation was given at three different dose rates (DR): Low (LDR 2.5 mGy/h), Mid (MDR 10 mGy/h), and High (HDR 100 mGy/h). All dose rate groups received a cumulative dose of 3 Gy; exposure duration for each exposure group in hours is stated in the figure. One day post-radiation, all three dose rate groups were profiled. For the Late effects, only LDR and HDR were included. The figure is modified from previously published material [36].

The chronic gamma radiation exposure was performed at the FIGARO low dose gamma irradiation facility (Norwegian University of Life Sciences, Ås, Norway) managed by the CoE Centre of Environmental Radioactivity (CERAD CoE). The mice were moved and acclimatized at the radiation facility before radiation started. This study includes two segments to obtain specific information regarding early (one day post-radiation) and late effects (>100 days post-radiation) of ionizing radiation given by different dose rates (Fig 1).

**Fig 2:**
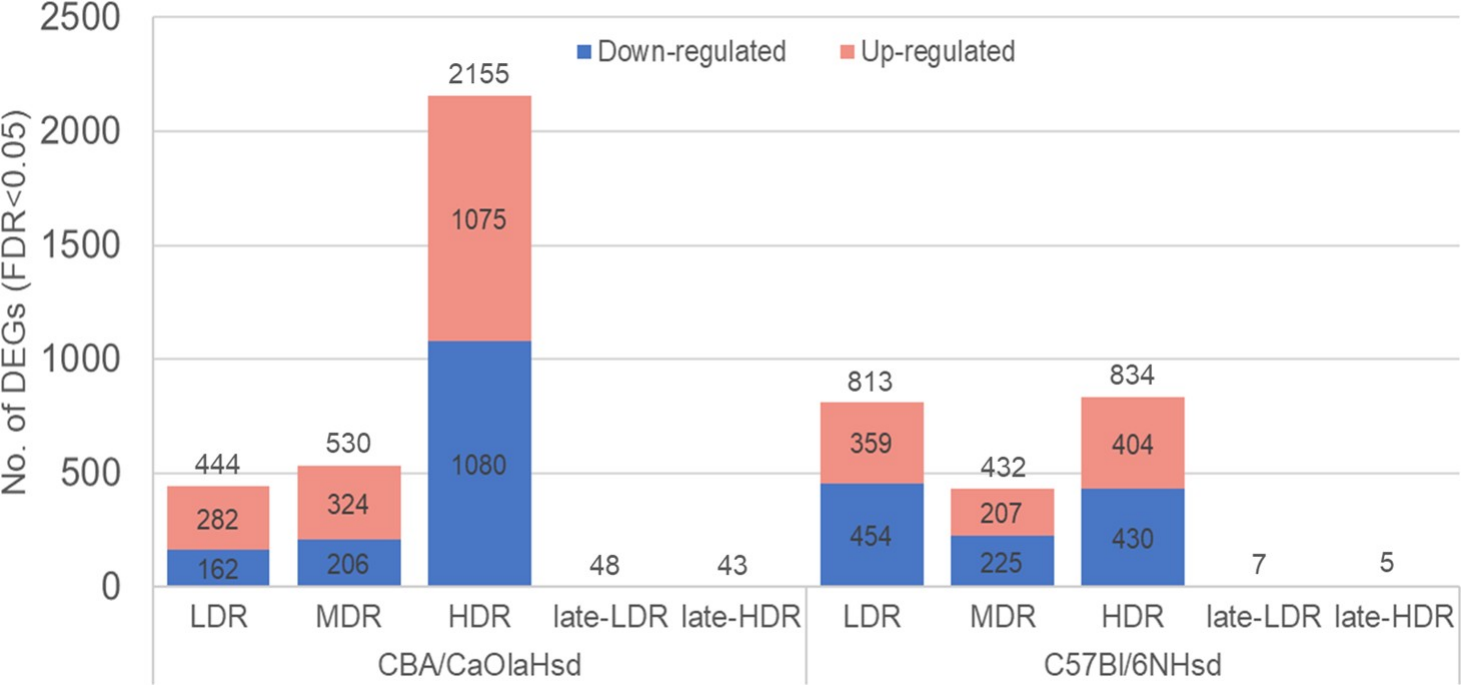
Numbers of differentially expressed genes. Total numbers of statistically significant (FDR<0.05) differentially expressed genes (DEGs) one day post-radiation (LDR, MDR, and HDR) and following recovery of >100 days post-radiation (late-LDR and late-HDR). The stacked bar plot indicates the numbers of significant DEGs (compared to control) for each dose rate and mouse strain (total number DEGs are seen over the bar; upper section (red): Number of up-regulated DEGs; lower section (blue): Numbers of down-regulated DEGs). For late-LDR and late-HDR, only total numbers of DEGs are given.

A total of 70 mice (35 CBA and 35 B6 mice) were used for the experimental setup. Groups of five mice (8–9 weeks at radiation start) were divided into 14 experimental study groups; control (early and late); low dose rate (LDR) (early and late); mid dose rate (MDR) (early), and high dose rate (HDR) (early and late). Each experimental exposure group included five mice. The same day as radiation ended, the mice were transported to the Norwegian Institute of Public Health (NIPH, Oslo, Norway) to terminate the early effect groups, housing of the recovery groups, and breeding inter- and trans-generational study groups. The early effect mice were terminated (age: 9–16 weeks, Fig 2) the day after irradiation ended (19–26 hours). The late effects mice were terminated 106–221 days post-radiation (106–221 days for B6 and 108–178 days for CBA; age range at termination 17–41 weeks). Age range and termination timepoints is reflected by the breeding regimes.

### Radiation and dosimetry

All exposure groups received a pre-calculated total dose of 3 Gy gamma radiation (^60^Co-source), given at different dose rates; 2.5 (LDR), 10 (MDR), and 100 (HDR) mGy/h. The pre-calculated exposure duration was 1200, 300, and 30 h, respectively. The dosimetry was performed using nanoDots [25,37]. The numeric value of air kerma to whole-body absorbed dose conversion coefficient for chronic exposures was 0.932 ± 0.008, resulting in a total whole-body absorbed dose of 2.60 ± 0.19 Gy for the 2.5 mGy/h-group, 2.67 ± 0.16 Gy for the 10 mGy/h-group and 2.65 ± 0.13 for the 100 mGy/h-group, all denoted as 3 Gy throughout the article. Irradiation was interrupted daily for 30–120 min for animal care. The beam-on time was adjusted correspondingly to achieve the pre-calculated exposure duration and hence total dose of 3 Gy. All cages were rotated daily to assure uniform exposure. Unexposed control mice were housed behind lead shielding outside the radiation field but within the exposure room.

### Global DNA methylation

DNA was isolated from CBA liver using the QIAamp DNA Mini kit (Cat.no. 51304). Integrity and concentrations were assessed in 2–4 technical parallels. Global levels of 5-methylcytosine (5mC) and 5-hydroxymethylcytosine (5hmC) were quantified using liquid chromatography-mass spectrometry/mass spectrometry (LC-MS/MS) as described in detail [38].

In brief, DNA is enzymatically digested to nucleosides, after which a mixture of internal standards was added. Pilot experiments were performed to establish a suitable mouse liver standard curve range at 0–5% for 5mC and 0–0.08% for 5hmC, all relative to guanine (G). A volume of 5 μL was injected on an Agilent 1200 μHPLC coupled with a triple quadrupole (QQQ) MS (6490, Agilent). The conditions for the LC/MS-MS analysis, calculation of sample concentrations, and quality control were as described [38]. A quality control sample composed of pooled DNA from human peripheral blood was included in every run to ensure that inter-run accuracy was within acceptable limits. 5mC and 5hmC are given as % of total cytosine (C) (represented by levels of the complementary G), and ratio 5mC:5hmC was calculated.

### RNA isolation

Total RNA was isolated from liver tissue using the miRNeasy mini kit (Qiagen, Hilden, Germany, cat. #217004) according to the manufacturer’s instructions. Lysis buffer was added to the frozen liver tissue and homogenized as previously described [36].

RNA quality and quantity were assessed using Agilent 2100 Bioanalyzer (Agilent Technologies, Santa Clara, California, USA), Qubit 2.0 with RNA-BR (Broad range) assay kit (Thermo Fisher Scientific, Massachusetts, USA), and a NanoDrop Spectrophotometer (Thermo Fisher Scientific, Massachusetts, USA).

### RNA sequencing

Library preparations (TruSeq Stranded mRNA kit (v2, Illumina) (insert length 250~300)) and paired-end sequencing (2 X 150 bp) were performed by Novogen Co., Ltd (Cambridge, UK). The early and late samples were sequenced in two separated laboratory setups.

### Quantitative real-time PCR

Quantitative real-time PCR (qPCR) was used to validate some selected statistically significantly differential expressed genes (DEGs) identified by RNA-Seq analysis, i.e., 29 genes (18 target genes (listed in S2 Fig) and 11 reference genes (*Araf*, *Cfl2*, *Coa5*, *Gapdh*, *Hprt*, *Mapk1*, *Pgk1*, *Rpl13a*, *Tbp*, *Vps54* and *Ywhaz*)). The reverse transcription reaction and qPCR analyses were carried out as previously described [39–41] In brief, total RNA (1.0 μg) from each sample was reverse transcribed to cDNA using the High Capacity cDNA Reverse Transcription Kit (Thermo Fisher Scientific, Massachusetts, USA) according to the manufacturer’s protocol. The resulting reverse transcription reaction product was stored at −20°C for further analysis.

Gene specific qPCR analysis was carried out in 384-well plates using QuantiTect SYBR Green PCR kit (Qiagen, Hilden, Germany) according to the manufacturer’s protocol, on a CFX384 Touch Real-Time PCR Detection System (Bio-Rad, Hercules, California, USA). cDNA (1:40 dilution) from each sample was run for each gene. All samples were analyzed on the same 384-plate to reduce run-by-run variations. A melting curve analysis and non-template controls (NTC) were included in each run. The quantification cycle (Cq) values were recorded with CFX Manager™ Software (Bio-Rad, Hercules, California, USA). One target gene was excluded from downstream analysis due to primer mismatch. The raw Cq values were analyzed by the comparative Cq–method [42,43] as previously described [39,40]. Target genes were normalized by the average of eleven reference genes by calculating ΔCq; where ΔCq (sample) = Cq (target gene) − Cq (mean of reference genes). The eleven reference genes were selected from RNAseq data based on their stability in both control and irradiated groups (CV% < 3%).

### Bioinformatic pipeline and functional gene enrichment

Raw reads (fastq files) were trimmed and filtered using Trimmomatic (version 0.39) using recommended settings (http://www.usadellab.org/cms/?page=trimmomatic). Trimmed reads were mapped to the mouse genome (version GRCm38; NCBI annotation) using HISAT2 (version 2.2.0.) [44] with default settings. Counting of mapped reads was done using the HTSeq-count program [45] (HTSeq package version 0.11.1.). Approximately 84x10^6^ reads were generated per library, and the average mapping rate was >95%. The counting of mapped reads with acceptable coverage generated more than 12.000 genes per sample. Raw count files have been made available via the NCBI Sequence Read Archive (SRA) (Accession: PRJNA747753).

### Statistical analysis

Differentially expressed genes (DEGs) were identified using the R (v. 4.0.1) wrapper SARTools [46] (v. 1.7.3) and EdgeR [47,48] (v. 3.86.1.) with default parameters (TMM normalization, cpm cutoff = 1). The false discovery rate (FDR) was set to 0.05 to identify the significant DEGs compared to controls. The log2-ratio (log2-FoldChange) was used to evaluate the level and direction of the expression.

Statistically significant DEGs, regardless of the log2-ratio, were compared to KEGG [49] and Gene Ontology (GO) [50,51] Biological gene sets implementing over-representations analysis (ORA) through EnrichR [52,53]. 8918 genes are members of a KEGG gene set, while 11 290 genes are annotated in GO Biological Terms gene sets (2021-04-26). DEGs common for all dose rates groups within strain was evaluated in EnrichR against the database “Transcription Factor (TF) perturbation followed by expression”, experiments mined from the Gene Expression Omnibus [54,55].

The differential gene expression was compared between strains at each dose rate by Pearson’s correlation and linear regression using CBA log2-ratios on B6 log2-ratios. Linear regression (F-test) were used to validate qPCR ΔCq-values and RNASeq log2-ratioes. Global methylation data were analyzed by one-way ANOVA using dose rate as independent variable and the Dunnett’s t-test as post hoc analysis. All statistical analysis has been performed using JMP Pro 15.2.0 (SAS Institute, NC, USA), and additional graphical illustrations were made using Office 360 and the R package *ggplot2* (https://ggplot2.tidyverse.org/).

## Results

Overall, we identified statistically significant changed hepatic transcriptional profiles, in both CBA and B6, for both the low (2.5 mGy/h), medium (10 mGy/h), and high (100 mGy/h) dose rate exposure groups one day post-radiation, when radiation was given to a similar total dose of 3 Gy. These observed transcriptional modulations included two main findings; I) dose rate specific response, seen by a) different numbers of DEGs, b) low overlap of DEGs, and c) difference in representation of the functional pathways, and II) strain-specific response, identified by differently modulated transcriptional response across dose rates. Evidence of changed epigenetic regulation, assessed by global DNA methylation, was not seen. After a recovery period of >100 days, the number of differentially expressed genes between treatment groups and controls was markedly lower.

### Differentially expressed genes in CBA and B6 one day post-radiation

**Fig 3:**
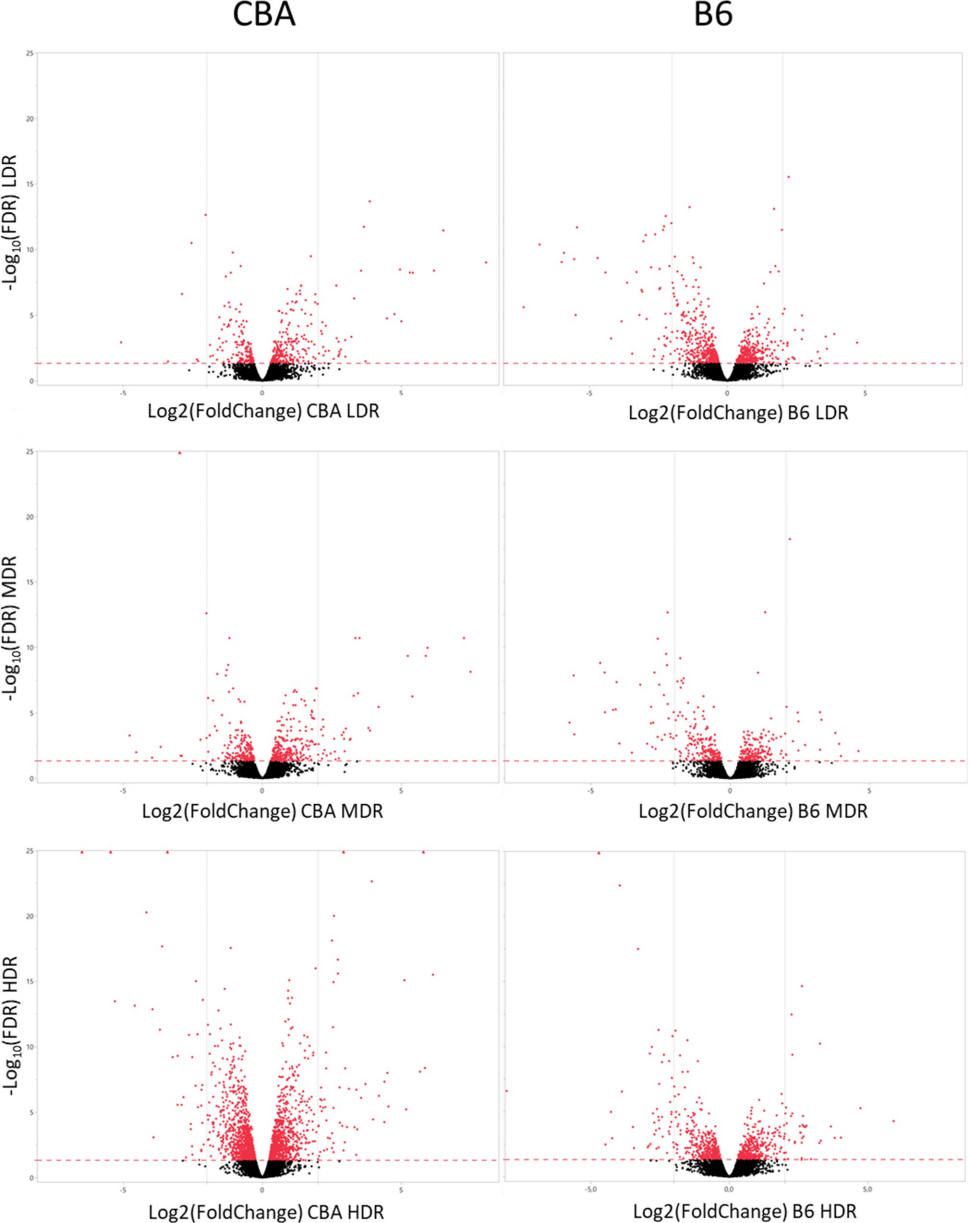
Volcano plots illustrating the radiation induced response on gene expression one day post-radiation in CBA and B6. The **s**tatistically significant threshold (FDR = 0.05) are shown by a read dashed line, and the statistically significant DEGs (FDR < 0.05) are shown in red. The vertical line represents log2-ratio = 2, and dots left and right of this line have log2-ratios > 2.

Exposure to chronic LDR induced statistically significant DEGs in both CBA and B6 (Figs 2 and 3). Comparing the numbers of significant DEGs for each dose rate, CBA showed a more pronounced increase in the numbers of DEGs, than B6 which showed comparable numbers of DEGs at LDR and HDR. Regardless of dose rate, CBA expressed 2608 significant DEGs in total compared to control one day after gamma radiation, whereas B6 expressed a total of 1449 significant DEGs. All identified statistically significant DEGs is presented in S1 Table.

The identified DEGs were correlated with previously identified and published miRNAs-markers [36] identified as possible predictors of exposure to γ-radiation. These miRNAs were identified from the same livers as used in this experiment. The correlation analysis was performed using Ingenuity Pathway Analysis (IPA) (Qiagen, Hilden, Germany - www.digitalinsights.qiagen.com), and is presented in S1 Fig. We observed some inverses correlation between six of the predicted miRNAs and a few of the genes in our DEGs list.

From the qPCR validation analysis of the selected DEGs, we observed a good correlation between the ΔCq-values and the RNA-Seq normalized counts (R^2^ = 0.806, p-value < 0.001) (S2 Fig).

**Fig 4:**
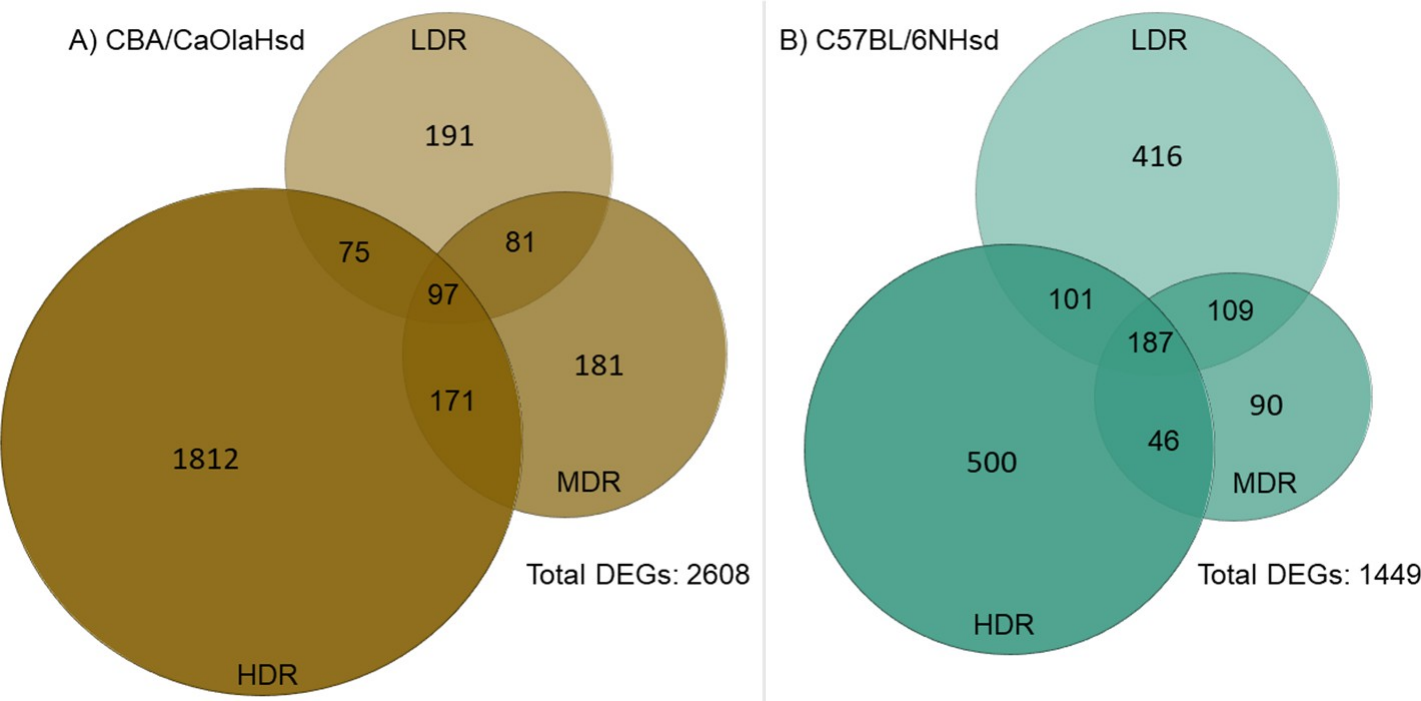
Venn diagrams of the differentially expressed genes. The Venn diagrams illustrate the numbers of statistically significant DEGs specific and overlapping across dose rate for the two strains: A) CBA/CaOlaHsd and B) C57BL/6NHsd.

Evaluating the overlapping DEGs across dose rate (Fig 4); CBA LDR and MDR shared 22%, MDR and HDR 11%, and LDR and HDR 7% of the DEGs. Comparing all three CBA dose rate groups, only 3% mutual DEGs were observed (Fig 4A). B6 showed an overall higher degree of common DEGs across dose rates, with 31% for LDR and MDR, 23% for MDR and HDR, and 21% for LDR and HDR. There were 9% mutual DEGs when all three B6 dose rate groups were compared (Fig 4B).

**Fig 5:**
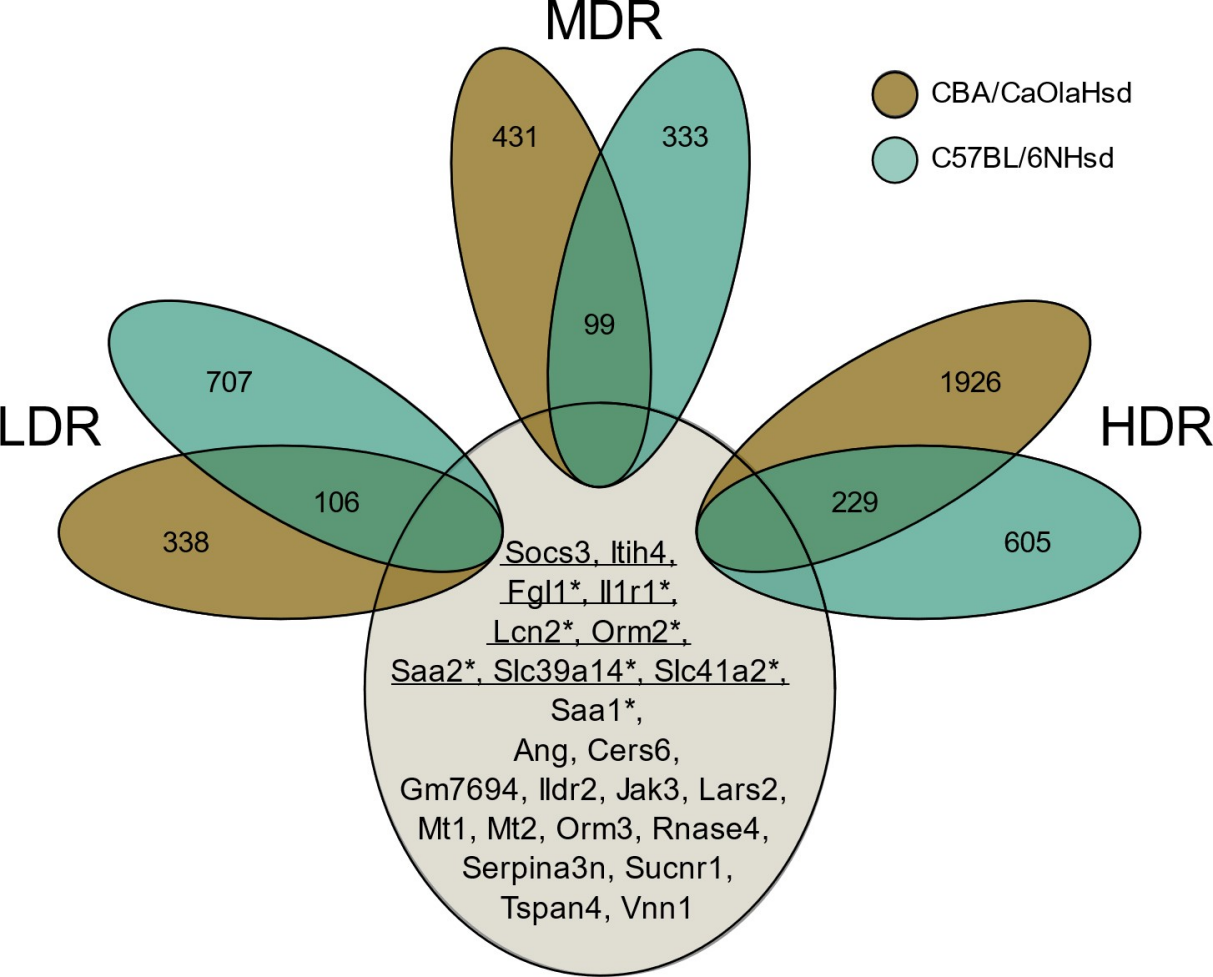
Venn diagram illustrating dose-rate-specific transcriptional features across strain. There were 24 DEGs common for all groups, indicated with gene names. Genes related to the transcription factor *Stat3* (eight genes) are underlined, and genes with a * are related to the transcription factor *Myc* (seven genes).

Comparing the statistically significant DEGs for each dose rate group across strain (Fig 5), we observed the following degree of mutual DEGs; LDR_CBA_LDR_B6_: 9%, MDR_CBA_MDR_B6_: 12%, HDR_CBA_HDR_B6_: 8%. Overall, 24 DEGs overlapped across all exposure groups (Fig 5). Out of these, 19 DEGs were inversely expressed in the two strains, i.e., upregulated in the CBA strain and downregulated in the B6 strain. The 24 common DEGs were evaluated using the database “Transcription factor perturbation followed by gene expression”. The over-representation analysis revealed DEGs with highly statistically significant enrichment to the transcription factors *Stat3* (6.882e-9) and *Myc* (1.197–8).

Evaluation of the strain-specific relationship for each dose rate showed a tendency for opposite expression levels in B6 compared to CBA for both LDR and MDR groups (β`s of -0.81 and -0.52, respectively). For HDR, no correlation (regression β = 0 and R-square = 0) was detected. This independence between DEG levels indicates a strong strain-specific response after the acute high dose rate exposure.

### Functional enriched pathways

**Fig 6:**
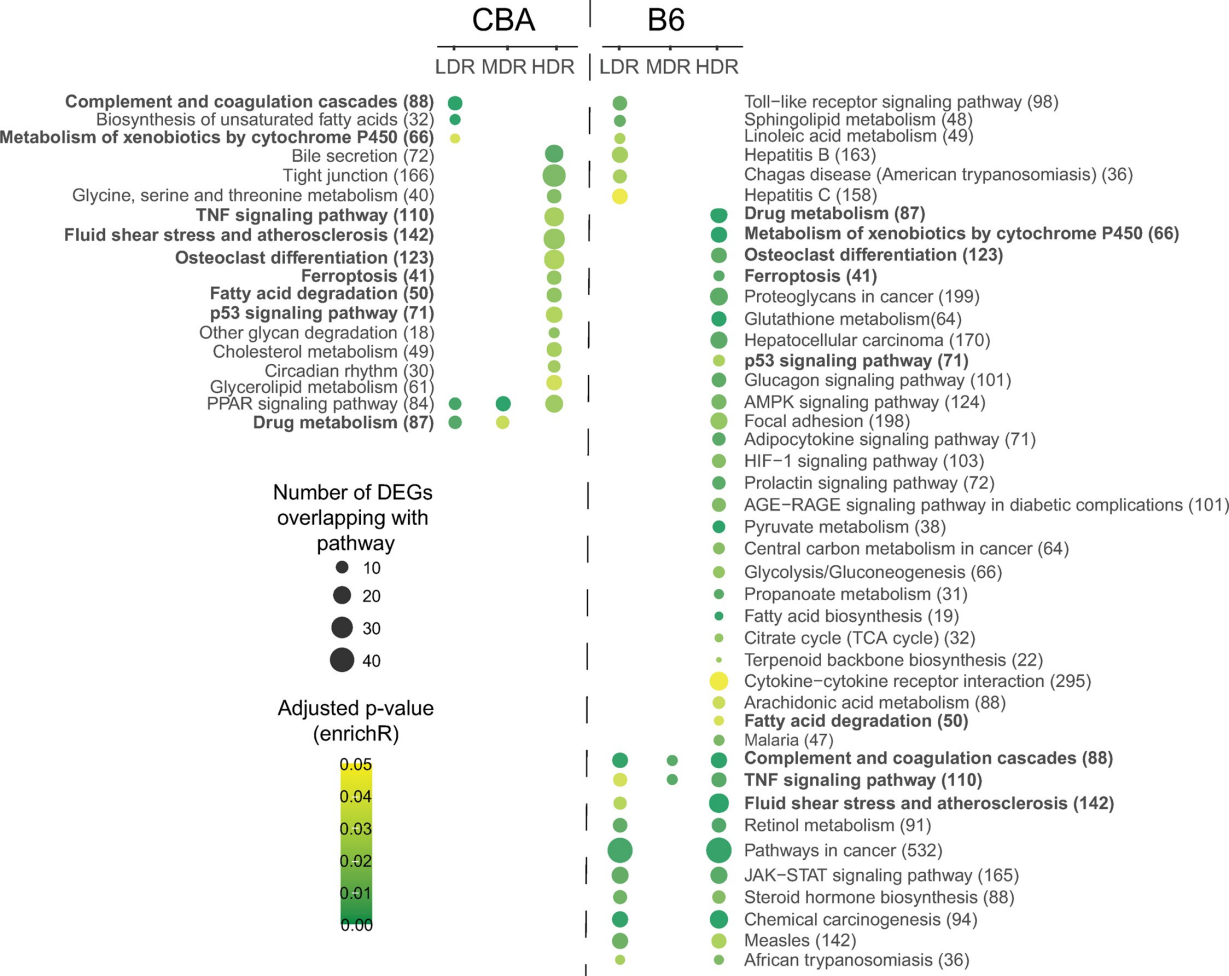
Functional pathway enrichment analysis visualized as a strain-specific dot-plot. The statistically significant (adj. p-value < 0.05) over-represented pathways for all exposure groups are shown. The pathways are sorted within stain by dose rate (LDR-MDR-HDR). Dot color indicates the level of significance, and dot size reflects the number of identified DEGs represented for the pathway. Pathways highlighted in bold are enriched in both strains. The total number of genes annotated to each KEGG gene set is shown in brackets.

Biological significance related to the identified DEGs one day post-radiation was evaluated using the gene sets available in the KEGG database. The statistically significantly over-represented pathways (adj. p < 0.05) for each dose rate per strain is presented in Fig 6. Of the identified DEGs, 10% (CBA) and 25% (B6) were annotated to a functional pathway. Even if CBA displayed a higher total number of statistically significant perturbed DEGs than B6, CBA displayed a lower number of statistically over-represented functional pathways compared to B6. For both strains, the number of DEGs associated with functional pathways for the different dose rates was in the order of HDR>LDR>MDR. Nine pathways were common for both strains regardless of dose rate, however not identified for the same dose rate groups across strain (Fig 6, indicated in bold). Overall, functional, and biological significance seen by the identified perturbed pathways is related to cancer, lipid metabolism, and inflammation.

#### CBA one day post-radiation

In CBA (Fig 6), five pathways were enriched for LDR, two for MDR, and fourteen for HDR. Three pathways were specific for LDR, whereas thirteen pathways were modulated only for HDR. The percentage of DEGs represented in a significant KEGG gene set for LDR, MDR and HDR were 9%, 5%, and 10%, respectively. The only pathway common for all dose rates was the “PPAR signaling pathway”. However, increasing the adjusted p-value to < 0.1, the “Biosynthesis of unsaturated fatty acid” appeared for all dose rates (MDR adj. p-value = 0.072 and HDR adj. p-value = 0.086). All DEGs mapping to “PPAR Signaling pathway” and “Biosynthesis of unsaturated fatty acid” were downregulated compared to the control.

For CBA LDR, the significant KEGG Gene Sets seen in Fig 6. indicated a response associated with the biosynthesis of fatty acids and the activation of complement. Due to multiple annotations across several biological processes, identical DEGs are seen for multiple KEGG Gene Sets; different DEGs of the Glutathione s-Transferase family overlap between “Drug metabolism” and “The metabolism of xenobiotics by cytochrome P450”. Most of the CBA LDR DEGs represented to a KEGG Gene Set shown in Fig 6. were suppressed compared to controls, except “Complement and coagulation cascade”, where all represented DEGs were upregulated. Using the GO terms for Biological Processes to generalize the functional representation of the DEGs (GO result output is presented in S2 Table), fatty acid biosynthetic process (GO:0006633), and regulation of complement activation (GO:0030449) confirm the findings in the KEGG database. Significant GO terms were also related to an acute inflammatory response (GO:0002673) and response to endoplasmic reticulum stress (GO:0034976).

A higher number of DEGs were significantly modulated in the CBA HDR exposure group and is reflected by a higher number of over-represented KEGG Gene Sets (Fig 6). Overall, the significantly perturbed functional pathways were mainly related to hepatic lipid metabolism as represented by several KEGG Gene Sets. Pathways associated with the regulation of cell survival and modulation of immune response were also perturbed; Ferroptosis (adj. p-value = 0.019), “p53 signaling pathway” (adj. p-value = 0.027), and “TNF Signaling Pathway” (adj. p-value = 0.024). The KEGG Gene Set “Fluid shear stress and atherosclerosis” were also significantly (adj. p-value 0.020) perturbed. However, whether the pathways are generally activated or suppressed is not clear based on these molecular patterns. Common DEGs were linked to the cytokine-related pathways “Osteoclast differentiation”, “TNF signaling pathway”, and “Fluid shear stress and atherosclerosis”.

#### In B6 one day post-radiation

Overall, in B6, fifteen pathways were enriched for LDR, three for MDR and 36 for HDR (Fig 6). Six pathways were specific for LDR, whereas 25 pathways were specific for HDR. The percentage of DEGs represented by a significant KEGG gene set were 14% for LDR, 6% for MDR and 23% for HDR. The KEGG Gene Sets, “TNF Signaling Pathway” and “Complement and coagulation cascade”, was perturbed for all dose rates.

In LDR, the KEGG Gene Sets identified from modulated DEGs after LDR exposure show perturbation in biological processes related to inflammation and cellular lipid metabolism (Fig 6). Some of these pathways are significantly expressed only for B6 LDR; the related inflammatory gene sets: “Toll-like receptor signaling pathway”, “Hepatitis B and C” and “Chagas disease”, and the response of cellular lipids: “sphingolipid metabolism” and “linoleic acid metabolism”. The KEGG gene sets related to “Hepatitis B and C” and “Chagas disease” mainly consisted of DEGs also represented by the toll-like receptor signaling pathway. Represented DEGs in the gene set for “linoleic acid metabolism” overlap with the gene sets for “retinol metabolism” and “steroid hormone biosynthesis”, gene sets commonly represented for both LDR and HDR. Using GO terms for biological processes to generalize the functional representation of the DEGs, GO terms related to both an inflammatory response and response to lipids appeared significant (S2 Table). The GO terms also revealed statistically significant representation of DEGs to gene sets downregulating apoptotic signaling (“Negative regulation of extrinsic apoptotic signaling pathway via death domain receptors” (GO:1902042), adj. p-value = 0.4e-3). The DEGs enriched to the regulation of apoptotic signaling was also seen in pathways related to endothelial cells (“Negative regulation of endothelial cell apoptotic signaling pathway” (GO:2000352, adj. p-value = 0.009)).

For HDR, which expressed a comparable number of significant DEGs as LDR (Fig 2), showed a higher number of significant over-represented pathways than LDR. These KEGG gene sets represent signaling pathways relevant for several functional mechanisms, ranging from carcinogenesis, inflammation, lipid metabolism and energy production. Exploring the GO biological process terms, DEGs are represented in gene sets related to the regulation of steroid biosynthetic processes (GO:0050810, adj. p-value = 3.12e-8), glutathione derivative biosynthetic processes (GO:1901687, adj. p-value = 3.8e-5), acute-phase inflammatory response (GO:0006953, adj. p-value = 7.0e-5) and several significant gene sets related to the regulation of apoptotic response.

#### B6 and CBA >100 days post-radiation

Transcriptional changes that persist after considerable time for recovery (100–200 days post-radiation) were analyzed in a separate sequencing run than one day post-radiation, only focusing on the LDR and HDR groups. The late response groups showed a considerably lower number of modulated genes compared to control groups, than the early response groups. CBA showed a higher number of DEGs for both LDR and HDR than B6 (Fig 2).

No significant over-representation of functional pathways was seen for the CBA LDR late DEGs. However, the KEGG pathway “Transcriptional misregulation in cancer” were represented with four genes mapping to the pathway (*Bcl6*, *Zbtb16*, *Aff1*, and *Dusp6*), but not significant (adj. p-value = 0.07036). For the CBA HDR late DEGs, no significant over-represented pathways nor identified GO terms were seen. Pathway enrichment was not conducted for the B6 late groups due to the low numbers of significantly modulated DEGs.

### Oxidative stress

**Fig 7:**
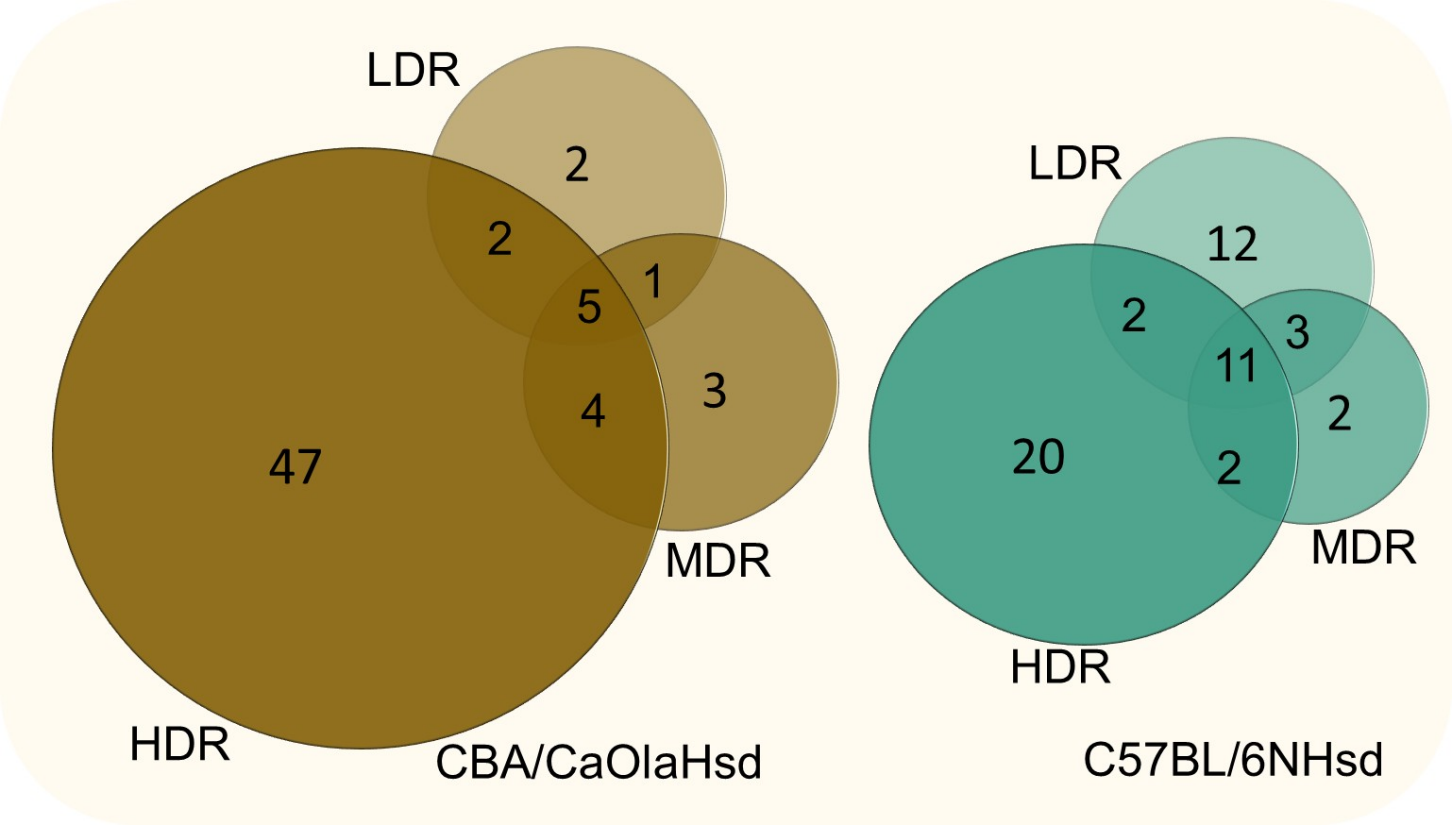
Oxidative stress related DEG. Numbers of DEGs overlapping with the GO Term “Response to Oxidative Stress” (GO:0006979 (408 annotated genes)) visualized with Venn diagrams for the two strains.

The KEGG database lacks a predefined pathway specific for genes related to oxidative stress (it only includes “oxidative phosphorylation” consisting of 134 genes). Due to this, and the fact that ionizing radiation is a potent inducer of ROS, we investigated how our significant DEGs overlapped with the Biological Process GO Term “Response to oxidative stress” (GO:0006979) consisting of 408 genes (Mouse Genome Informatics, www.informatics.jax.org, date:11.2.2021). The numbers and the overlapping DEGs are illustrated in Fig 7, and the identified oxidative stress-related genes are listed in the S1 Table.

In CBA, the number of significant DEGs increased with dose rate, and five genes overlapped for all dose rates. The LDR group displayed in total seven genes. Two of these genes were specific for the CBA LDR group, *Aldh3b1* (Aldehyde Dehydrogenase 3 Family Member B1, adj. p-value = 1,34E-02) and *Cflar* (Casp8 And FadD Like Apoptosis Regulator, adj. p-value = 1,60E-02), both upregulated. In B6, eleven genes were mutual for all dose rates, and twelve genes were specific for LDR.

Functional enrichment analysis (not performed for LDR due to low numbers of genes) of the identified CBA HDR DEGs (58 genes) revealed a highly statistically significant GO term “Response to hydrogen peroxide (GO:0042542)” (adj. p-value = 6.251e-10), a reactive oxygen species known to be induced by ionizing radiation. Among the KEGG Gene Sets “Apoptosis” (adj. p-value = 1.39e-5), “Fluid shear stress and atherosclerosis” (adj. p-value = 1.39e-5), “Ferroptosis” (adj. p-value = 2.04e-4), “Mitophagy” (adj. p-value = 7.24e-4), and “Protein processing in ER” (adj. p-value = 0.0021) were significantly over-represented. In B6, exploring the specific oxidative stress related LDR DEGs (12 genes) in the KEGG database, several pathways were statistically enriched. The genes *Jun*, *Atf4*, and *Egfr* were annotated to several of these functional pathways. Evaluating all significant DEGs identified for B6 LDR (28 genes), “Apoptosis” is the most significantly enriched pathway (adj. p-value = 4.8e-6). Considering the oxidative stress-related HDR B6 DEGs (35 genes) with the KEGG database, “Fluid shear stress and atherosclerosis” (adj. p-value 1.614e-8), “Apoptosis” (adj. p-value = 1.09e-5) and “Pathways in cancer” (adj. p-value = 1.49e-4) were the three most significantly enriched pathways.

Across the two strains, two DEGs were shared: Vanin-1 (*Vnn1*) and Lipocalin-2 (*Lcn2*). *Vnn1* was suppressed for all exposure groups in both strains, whereas *Lnc2* was upregulated in CBA and downregulated in B6. However, *Lnc2* displayed a high individual variation in expression level.

### Global DNA methylation

**Fig 8:**
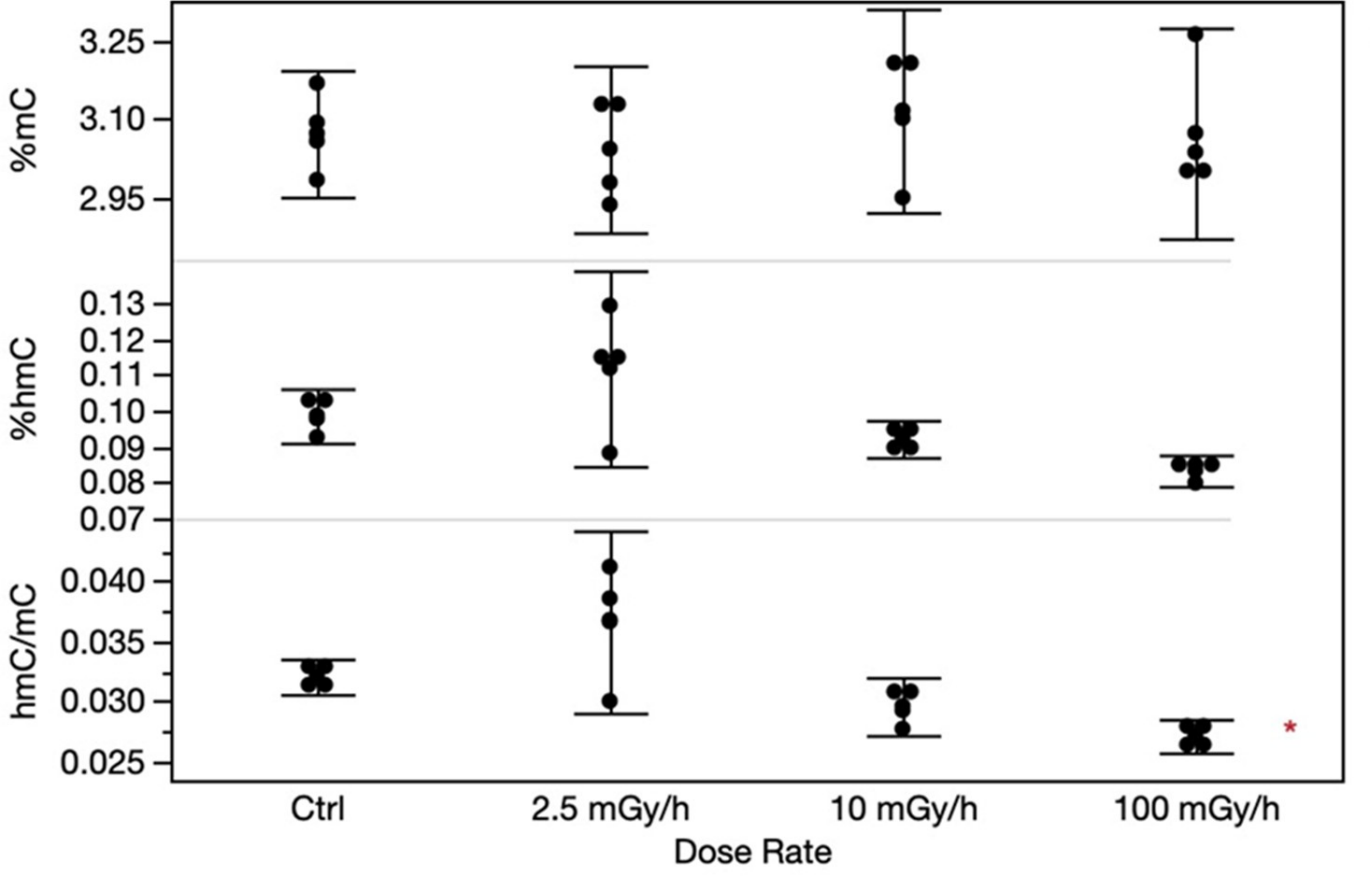
The levels of global methylation in CBA/CaOlaHsd livers analyzed by HPLC-MS/MS. In the panels, %mC (upper panel) and %hmC (mid panel), and the ratio between %hmC and %mC (lower panel) are shown. Each error bar represents the 95% confidence intervals of the means. Asterisk indicate borderline significance (p = 0.043) for the HDR hmC:mC-ratio.

Gene expression is regulated by epigenetic mechanisms, hence we assessed global DNA methylation levels of both 5mC and 5hmC in CBA livers using HPLC-MS. The levels of 5mC and 5hmC did not change significantly due to the exposure to ionizing radiation at any dose rate level, however the 5mC:5hmC-ratio were borderline significant (p = 0.043) for the HDR exposure group using Dunnett`s test (Fig 8). However, applying Tukey`s test with multiple corrections, this significant level disappears.

## Discussion

Insights into molecular events initiated by exposure to different dose rates can give valuable contributions to the ongoing debate regarding the relevance of the dose rate upon cellular responses and health outcomes of ionizing radiation [24,32,56]. Our study shows that hepatic transcriptional profiles are modulated **dose-rate-specific** in response to whole-body exposure to an equal total dose gamma irradiation, given at low, medium, and high dose rates, chronic to acute. The study also demonstrates that the transcriptional response is modulated differently for CBA and B6 mice, indicating **strain-specific** irradiation-induced responses.

The chronic low dose rate used in our study (2.5 mGy/h; ~60 mGy/day for 55 days (3 Gy)) initiated transcriptional events in both CBA and B6 livers. Our results for the LDR exposure group are in line with another study using chronic low dose rate exposure, reporting modulation to the B6 hepatic gene expression profile [57]. However, compared to our study, the dose rates were lower (20, 1.0, and 0.05 mGy/day), and the total doses were not directly comparable (8.0, 0.4, and 0.02 Gy). Collectively, the results demonstrate that ionizing radiation given at chronic low dose rates does initiate molecular events, suggesting that such exposures may impact response cascades important for radiation-induced biological outcomes.

The transcriptional profile, one day post-radiation, differed between the two mouse strains (Figs 2–5). Within CBA mice, the group exposed to low dose rate shared only 7% of the DEGs with the high dose rate. The corresponding number in B6 mice was markedly higher, 20%. These dissimilarities are reflected in the pathway enrichment analysis that revealed differing results depending on both strain and dose rate (Fig 6). Recently, radiation-induced strain-specific gene expression response was reported after acute irradiation of BALB/c and CBA mice [58]. Strain-specific radiation-induced responses have also been reported from others investigating other endpoints than gene expression [59–61], suggesting C57BL/6 be less susceptible to radiation-induced responses. Together with our results, we conclude that mouse genetic background is important when evaluating low LET radiation-induced genetic instability [30].

In general, the biological significance of the modulated DEGs in each exposure group revealed pathways involved in cancer, lipid metabolism, and inflammation. One day post-radiation, DEGs involved in cancer development and suppression, as well as ROS, were identified after HDR exposure in both strains: i.e., “*p53 signaling pathway”* and “*Ferroptosis”*. Ferroptosis is intriguing in this context. It is an iron-dependent form of programmed cell death induced by accumulation of lipid peroxidation (mainly peroxidation of phospholipids), resulting from an overload of the protective glutathione-dependent antioxidants and characterized by mitochondrial shrinkage [62]. Additionally, in B6, the HDR exhibit “*Pathways for cancer”*, *“Chemical carcinogenesis”*, *“Proteoglycans in cancer”*, and *“Hepatocellular carcinoma”*. Exposure to high dose rates of ionizing radiation to the total dose used herein is acknowledged to increase cancer risk. Gene expression changes indicating carcinogenic effects for the HDR exposure group were anticipated. “*Pathways for cancer”* and *“Chemical carcinogenesis”* were also significantly perturbed for the B6 LDR exposure group, suggesting that exposure chronically to a low dose rate, although given to a high total dose of 3 Gy, do modulate genes involved in cancer development. The majority of the DEGs represented in “Chemical carcinogenesis” were upregulated, while the DEGs represented in “Pathways for cancer” were both up- and downregulated. Likewise, others have shown hepatic DEGs grouped to tumorigenesis and chromosomal damage in B6 mice following exposure to a low dose rate of 20 mGy/day to a total dose of 8 Gy. However, these genes were suppressed [57]. Regarding mutation induction, studying heteroplasmy in a mitochondrial gene (*Mtcyb*) in B6 and BALB/c exposed to the Chernobyl environment for 30–40 days (~0.04 Gy/day) receiving 1.2–1.6 Gy, showed no significant gene mutation risk [27]. Considering these studies, chronic low dose rate gives rise to molecular changes associated with cancer. The potential of ionizing radiation to impact biological effects appears to depend on both the dose rate and duration of exposure when the cumulative dose is kept constant.

Collections of genes related to lipid metabolism are dysregulated in both LDR and HDR exposure groups one day post-radiation. In CBA, the *“PPAR Signaling Pathway”* was perturbed for all three dose rates. The PPAR Signaling Pathway is involved in energy homeostasis, including cholesterol and fatty acid metabolism. PPARα has a central role in lipid (fatty acid oxidation) and glucose metabolism, where it exerts both an anti-inflammatory and an anti-oxidative function [63]. Elaborating on the specific genes represented in this pathway, the LDR group showed enrichment of PPARα target genes related to fatty acid transport and fatty acid oxidation. The numbers of DEGs enriched to fatty acid transport and oxidation increase in the higher dose rate groups. Whether the mechanisms, as such, are activated or suppressed remains unclear. Transcriptional change in the gene coding for the nuclear receptor PPARα itself was not observed at our sampling timepoint. In B6, other pathways related to lipid metabolism (i.e., fluid shear stress and atherosclerosis) were enriched for both LDR and HDR. However, not the PPAR signaling pathway. Previous findings of changed expression of genes related to hepatic lipid metabolism following low dose rate radiation are reported in both mice [57] and rats [64]. Together, the results suggest that exposure to low dose rate ionizing radiation perturb molecular signaling pathways related to different aspects of hepatic lipid metabolism, which may have implications for radiation-induced non-cancer effects such as cardiovascular disease and non-alcoholic fatty liver disease. In the B6 LDR group, other sets of genes related to the metabolism of sphingolipid and linoleic acid were significantly enriched. Sphingolipids are highly bioactive membrane lipids controlling cell division and cell death. Due to a high metabolic network among various sphingolipids, transcriptional modulation of genes related to the pathway *“sphingolipid metabolism”* could result in the progression of several conditions, i.e., diabetes and hepatocellular carcinoma [65].

Pathways related to aspects of inflammation are enriched for both LDR and HDR in both CBA and B6. In B6, perturbed DEGs are seen for the *“Toll-like receptor signaling pathway”*, which plays a crucial role in the innate immune system. The toll-like receptors initiate inflammation by recognizing damage-associated molecular patterns (DAMPs), molecules released by cell injury/death, and stress [66]. The *“Complement and coagulation cascade*” is perturbed in the CBA LDR exposure group and in all B6 dose rate groups. The toll-like receptors and the complement system are both players in the innate immune system. The crosstalk between them is related to the pathophysiology of atherosclerosis [67]. It is also known that the complement response is elicited following high acute doses such as those used in cancer therapy [68]. The perturbation of these two pathways by chronic LDR ionizing radiation suggests that inflammatory responses are elicited, which may contribute to the development of cardiovascular disease, including atherosclerosis, where ROS is known to play a central role. It should be kept in mind that the gene expression profiles observed may be an indirect effect of biological response in other tissues due to the whole-body exposure regime. The liver acts as a surveillance and response hub for the whole body by regulating systemic metabolism and maintaining homeostasis after external stimulus, particularly related to inflammatory responses and vascular damage [69,70].

Interestingly, we identified a gene set of 24 DEGs that could be representative of a radiation-specific transcriptional response, regardless of dose rate and genetic background (Fig 5). Transcription factor over-representation analysis revealed that the 24 genes were significantly associated with the transcription factors *Stat3* and *Myc*. *Stat*3 has been shown to be activated by gamma radiation *in vitro* [71]. In addition, upregulation of *Stat3* has been shown to have both anti- and pro-inflammatory roles during the pathogenesis of liver fibrosis [72]. *c-myc* has been linked with both ROS formation and DNA damage [73]. In our study, the *c-myc* transcription level was not differentially expressed in any of the exposure groups. On the other hand, the transcript level of *Stat3* was upregulated in CBA LDR and MDR and downregulated in B6 at all dose rates.

One of the major pathological routes of ionizing radiation toxicity is the generation of ROS [74,75]. We previously demonstrated induction of DNA damage in mice following similar and lower chronic low dose rates given to equal or lower total doses of gamma irradiation [25,76]. However, we do not have explicit evidence that these are indeed oxidative DNA lesions. Oxidative stress is a complex and multifactorial process resulting from an imbalance between ROS production (endogenic and exogenic production) and antioxidant capacity. Disturbance of redox homeostasis is linked to several pathological conditions, such as cardiovascular disease, hepatic damage [77], and tumor development [78]. Among the LDR and HDR enriched KEGG gene sets (Fig 6), no direct response to increased ROS generation or induction of DNA damage response was evident. One explanation could be that the sampling timepoint was sub-optimal regarding the transcriptional biodynamic of the genes in question. Several of the enriched functional pathways, for both LDR and HDR, could be associated with an imbalance in ROS homeostasis, even if it was not directly apparent in the identified transcriptional pathways.

Based on this, we performed pathway enrichment analysis on the genes annotated to the GO biological process “*Response to oxidative stress*” using the KEGG database. This exercise identified functional signaling pathways enriched with ROS annotated genes. Several of these “ROS-enriched” pathways were also enriched by our identified DEGs for all dose rate groups, regardless of the low representation of the ROS annotated genes. Higher numbers of specific oxidative stress-related DEGs were expressed in the HDR group than in the MDR and the LDR groups (Fig 7). In agreement with our HDR results, rats exposed to acute high dose rates of gamma radiation (1.5–3.5 Gy) once a week for one month expressed significantly changed antioxidant enzyme activities in liver and skeletal muscle tissues one month after radiation, which is indicative of oxidative stress [79]. Whether exposure to chronic low dose rate ionizing radiation in our study has led to an imbalance between the ROS generation and the antioxidant capacity, reaching a state of liver oxidative stress, is unclear. These results, taken together, suggest that exposure to chronic low dose rate ionizing radiation has a lower potential to bring ROS homeostasis to imbalance than higher dose rates, even if the total cumulative dose is equal and high. This is possibly linked to several factors, including the number of generated ROS per time unit of exposure and the cellular antioxidant capacity.

Among the identified ROS-related DEGs, two genes were modified across all groups: Vanin-1 (*Vnn1*) and Lipocalin-2 (*Lcn2)*. Vnn1 is highly expressed in centrilobular hepatocytes, and its function is related to energy production, coenzyme A-, lipid- [80], and xenobiotic metabolism [81]. Vnn1 is a tissue sensor of oxidative stress [82,83] and participates in stress-related adaptive tissue responses [84]. Vnn1-deficient mice exposed to whole-body gamma radiation (6 Gy acute) showed increased resistance to oxidative injury in the thymus compared to wild-type controls. The transient increased thymic expression of Vnn1 was seen two days post-radiation followed by down-regulation [85]. The observed down-regulation of Vnn1 in all dose rate groups in CBA and B6 in our study suggests that this gene could be an essential player in response to ionizing radiation, also after chronic low dose rate exposure.

To investigate the long-term effect of radiation, we measured changes in transcriptional responses after a recovery period of 100–200 days (14–28 weeks). The transcriptional response was, as expected, markedly lower in both strains, as the role of the transcriptional change is to rapidly and most often transiently respond to a cellular stress to ensure cellular integrity. The late_DEGs overlapped poorly with the one day post-radiation DEGs. The larger variation in age at sampling in the late effect recovery groups, due to breeding, could have impacted the individual variation in gene expression and hence affected the degree of significant DEGs. A recent report investigating long-term effects following acute exposure to gamma radiation (1 Gy) in CBA and BALB/c mice found alterations of hepatic gene expression profiles in BALB/c up to ten weeks post-radiation, whereas significant changes were identified in CBA/Ca only at four hours post-irradiation but not at later time points (24 h, one week and ten weeks) [58]. This is in line with the transcriptional response being mainly transient and not persist long-term. However, genes may be regulated on a longer time perspective to facilitate a rapid response in case of future exposures.

It has been proposed that exposure to chronic low dose rate radiation is more likely to induce epigenetic changes than acute exposures [86]. In our study, approaching changes in the epigenome at the global level did not reveal significant changes in the overall 5mC level or the 5hmC at the dose regimens tested (Fig 8). Methods investigating epigenetic mechanisms at base resolution level can be used in follow-up experiments to further explore the transcriptional etiology and other epigenetic mechanisms. For example, since epigenetic modulations could affect the transcription through changes in chromatin accessibility, assessing changes in chromatin architecture could reveal relevant epigenetic modulations initiated by ionizing radiation at the different dose rates.

## Conclusion

To our knowledge, this is the first study evaluating the impact of dose rate on the hepatic transcriptional profile after exposure to ionizing radiation in two mouse strains, in a chronic low dose rate versus acute high dose rate regimen to a similar total dose. We demonstrate that the transcriptional profile is differently modulated following low dose rate versus high dose rate exposure, illustrating the importance of both the dose rate, duration of exposure and total dose when evaluating radiation-induced molecular responses. As also discussed by others, our data, although at the level of gene expression, support that the cumulative dose alone may be insufficient to predict the associated risk, as both dose rate and duration of exposure seem to play a role. Exposure to chronic low dose rate ionizing radiation affects the maintenance of genomic and cellular integrity differently than acute high dose rate exposures. The marked differences in response between the mouse strains suggest variations in radiation-induced defense mechanism capacities. Our findings contribute to the understanding of radiation-induced carcinogenesis as well as non-cancer effects such as cardiovascular effects.

## Supporting information

S1 Fig Correlation between predicted miRNAs and identified DEGs. The table presents log_2_(FoldChange) values for six miRNAs predicted in Duale et al. (2020) that show correlations with some of our identified mRNAs. Inverse correlations are indicated with log_2_(FoldChange)-values **in bold**. Green upwards arrows indicate upregulations, while red downwards arrows indicate downregulation. Yellow arrows indicate mRNA expression levels less than the chosen log_2_(FoldChange) cutoff at 0.5.
(TIF)

S2 Fig Validation of DEGs using qPCR. Each dot or circle represents the ΔCq-value from the qPCR analysis and the log_2_[normalized count] from the RNA-Seq analysis of the selected targets analyzed for B6 control (circle) and B6 LDR samples (dot). The selected genes of interest are listed to the right.
(TIF)

S1 Table Excel file containing all identified statistically significant DEGs. DEGs representing one day post-radiation are found in the sheet: “DEGs_one_day_post_rad”, the late response is seen in sheet: “DEGs_>100days_post_rad”, and the identified oxidative stress related DEGs can be found in the sheet:”Ox_Stress_DEGs”. DEGs are listed by Gene_name, log_2_(FoldChange), and FDR.
(XLSX)

S2 Table Excel file containing the total result output using the GO biological process terms. (XLSX)
