# Supplementary figures and images for: Perturbed transcriptional profiles after chronic low dose rate radiation in mice
Source: PLoS One. 2021 Aug 24;16(8):e0256667. doi: 10.1371/journal.pone.0256667 (PMC8384182; doi:10.1371/journal.pone.0256667)

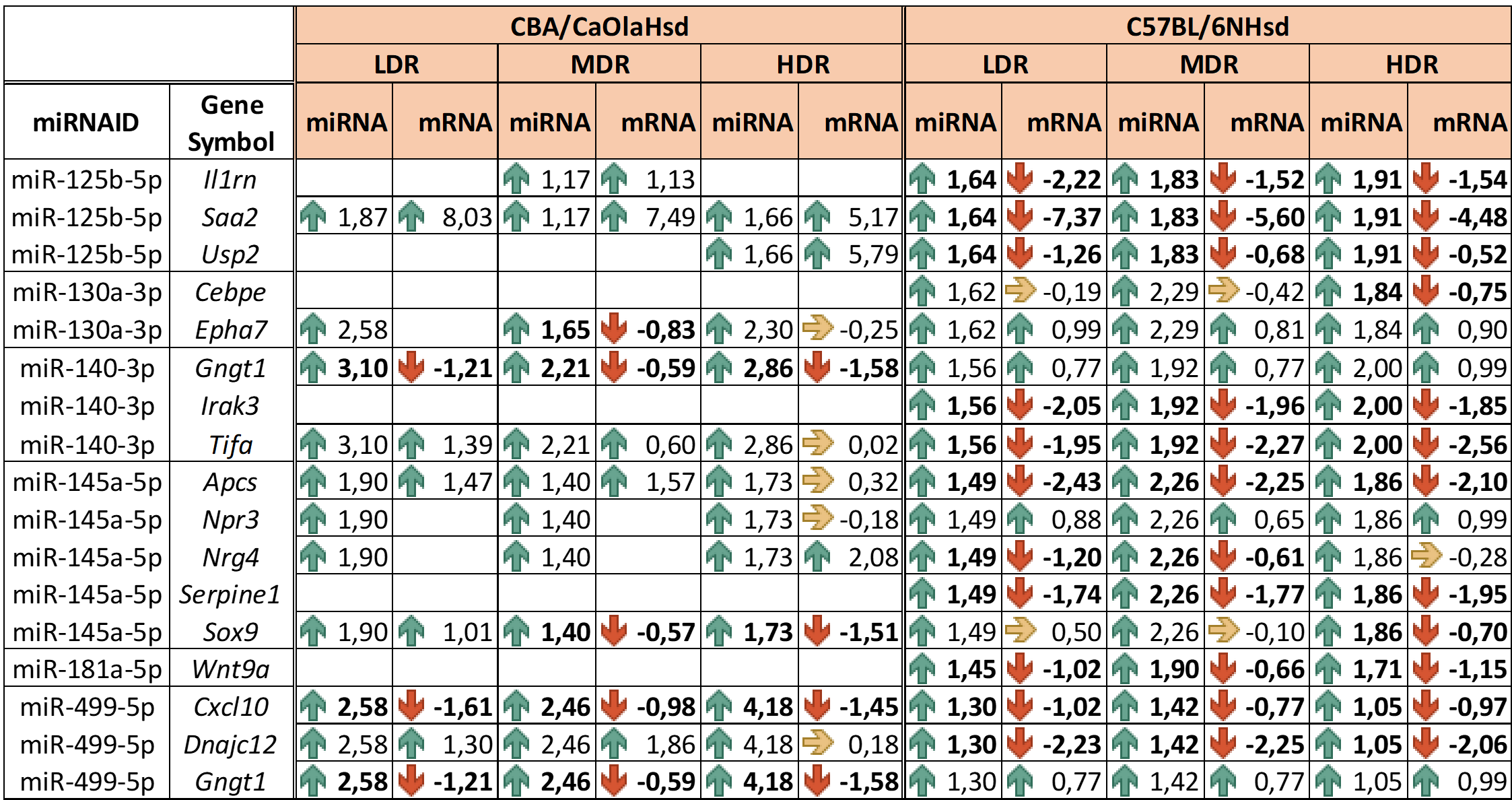

Supplement: S1 Fig — The table presents log2(FoldChange) values for six miRNAs predicted in Duale et al. (2020) that show correlations with some of our identified mRNAs. Inverse correlations are indicated with log2(FoldChange)-values in bold. Green upwards arrows indicate upregulations, while red downwards arrows indicate downregulation. Yellow arrows indicate mRNA expression levels less than the chosen log2(FoldChange) cutoff at 0.5. (TIF) [file pone.0256667.s001.tif]

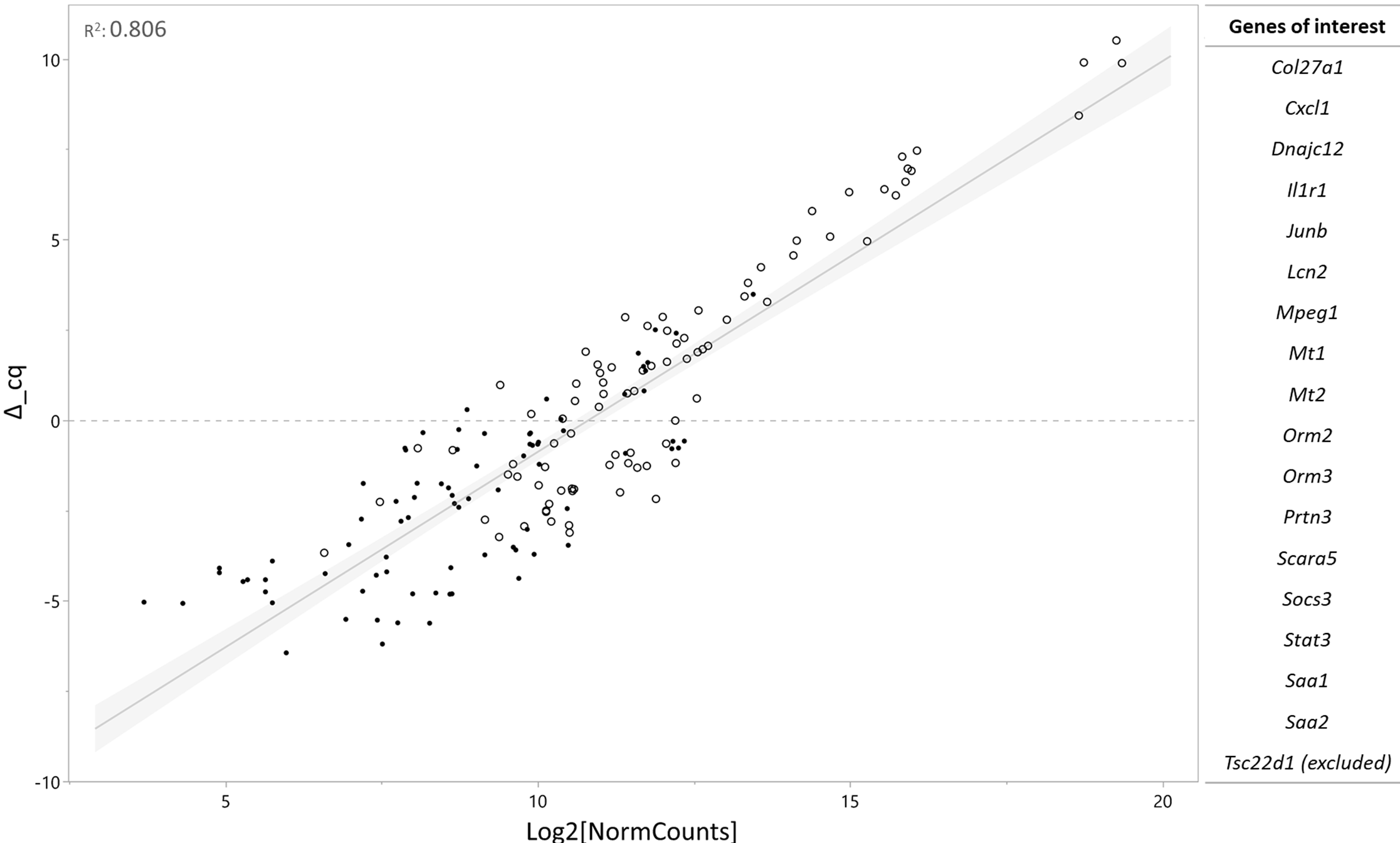

Supplement: S2 Fig — Each dot or circle represents the ΔCq-value from the qPCR analysis and the log2[normalized count] from the RNA-Seq analysis of the selected targets analyzed for B6 control (circle) and B6 LDR samples (dot). The selected genes of interest are listed to the right. (TIF) [file pone.0256667.s002.tif]
