# Supplementary material for: Perturbed transcriptional profiles after chronic low dose rate radiation in mice
Source: PLoS One. 2021 Aug 24;16(8):e0256667. doi: 10.1371/journal.pone.0256667 (PMC8384182; doi:10.1371/journal.pone.0256667)
Supplement: Attachment — Submitted filename: Response_to_Reviewers.docx [file pone.0256667.s005.docx]

# Response to editor and reviewers

**Comments to reviewers:**

Please note that the revised manuscript has changed page, line, and figure numbering and is no longer following the previous indications. In addition to the revisions based on the reviewers’ and requests, minor text editing has been made throughout the manuscript to ensure clarity and accurate grammar. All changes are tracked, and the line referencing is related to the manuscript with tracked changes.

**Response to editor’s comment:**

*"...suggestion #4 from reviewer #1 might require some additional work: "4.) Confirmation of the RNA-sequencing via alternative non-high-throughput measurements (qRT-PCR, protein expression) would enhance these results." Is this feasible? Perhaps this has already been done? Perhaps you don't feel this is necessary?”*

Thank you for this comment. We have now added results from a qPCR validation analysis of a selection of genes to the manuscript. For further details, see comment #4, reviewer #1.

------------------------------------------------------------------------------------------------------------------------------------

**To reviewer #1:**

**Thank you for your comments and for taking time reviewing this manuscript.**

Reviewer #1: The authors provide a novel hepatic transcriptional study with a chronic low dose rate in two mice strains at 24 hours and >100 days post-radiation exposure. The radiation endpoint was chosen to deliver the same total dose (3Gy) and provides an interesting direct comparison of dose-rate effects. The manuscript revealed a number of DEGs at 24 hours post-radiation exposure in both mice strains and discussed several functional pathways through enrichment analyses. However, at the second timepoint (>100 days) there were few DEGs and interpretation of functionally relevant pathways was limited. This may be expected given the long recovery time post-exposure, but is also confounded by the large time range when the mice were terminated (106-221 days). There was a brief comment about “breeding inter- and trans-generational study groups ”(line 142) in the methods section that may have influenced this result. While the authors also measured and found no statically significant changes in global DNA methylation, the results of this study are solely based on bioinformatic and statistical analysis of DEGs from RNA-sequencing. The strain-related differences/similarities in genes and pathways that were statistically significant add to the previously ascribed radiation resistance of the C57BL/6 strain found at higher doses and dose-rates.

1.) It would be useful to display the DEGs for each condition as a volcano plot to quickly visualize the fold-changes in relationship to the significance rather than referring to the supplemental data table. A hierarchical clustered heat map for the total DEGs per mouse strain (2,608 for CBA and 1,449 for B6) may also provide a useful alternative.

**Response to comment #1.**

Thank you for your suggestion for better visualization of the data. A volcano plot for each condition one day post-radiation has been added to the manuscript; **named Fig 3, the caption is found in lines 277 – 280, page no: 12.** All the following figure numbering are accordingly changed.

*2.) The authors state “no log2FC cut-off value was used” (line 198) when determining the direction of expression for the gene expression. However, based on the supplemental data provided, there was an inherent cut-off perhaps due to using an FDR <0.5.*

**Response to comment #2.**

Thank you for noticing the unclear description in the section for statistical analyses. The section for “statistical analysis” has now been clarified as modifications are made to the text: **lines 218 – 249, pages no: 9 – 11**.

3.) Are there any connections to the DEGs uncovered in this study and the related study on miRNAs perturbed by chronic low-dose irradiation (Duale et al. 2020)? While that study was examining biomarkers of LDR irradiation, any connections might strengthen the results found in the transcriptional study.

**Response to comment #3.**

Thank you for this suggestion. We have included a correlation analysis in determining an interaction between the predicted miRNA in Duale et al. 2020 and our identified genes. A figure has been added as **supplementary: S2 Figure**. The correlation analysis is mentioned in the result section, **page no. 12, lines 304 - 311.**

4.) Confirmation of the RNA-sequencing via alternative non-high-throughput measurements (qRT-PCR, protein expression) would enhance these results.

**Response to comment #4.**

We have now performed validation of a selection of DEGs using qPCR. A correlation Scatter plot has been added to the manuscript as **Supplementary: S3 Figure.**

The qPCR data are in high concordance with the RNA-Seq data. Hence, we consider that the RNA-Seq data performed at high sequencing depth (85 million reads per sample) gives reliable results, rendering a full qPCR confirmation redundant. After thorough considerations, we concluded that verification of **all** the statistically significant DEGs is unnecessary.

As these modifications require adding additional information to the section “material and method”, the subsection "Quantitative real-time PCR" has been added to **page no. 8, line 187 to page 9, line 207**.

The validation is reported in the result section, **page no. 12, lines 312 - 313.**

*5.) A limitation of pathway enrichment analysis is the dependence on predefined gene sets which led the authors to focus on the Biological Process GO Term “Response to oxidative stress” (GO:0006979) rather than relying on the KEGG database which lacks an ROS category. An alternative may include analyzing the current dataset with the Molecular Signatures Database (MSigDB) and performing a Gene Set Enrichment Analysis (Subramanian et al., Gene set enrichment analysis: A knowledge-based approach for interpreting genome-wide expression profiles, PNAS, 2005). This database includes many genesets other than KEGG and GO that may support the current analysis.*

**Response to comment #5.**

Thank you for this comment. The sub-section of “Oxidative stress” under “Results” has now been clarified as modifications are made to the text: **page 18, line 412 - 421.**

In the process of analyzing and evaluating our RNASeq data, a range of different bioinformatic strategies and enrichment tools were tested and evaluated (over-representation (ORA), ranking, and topological), including GSEA. EnrichR, as GSEA, includes a wide range of gene set libraries (also MSigDB). However, the statistical calculations are more simplified than GSEA, as EnrichR uses hypergeometric testing.

However, predefined gene sets related to a ROS category were not significant when evaluating output from different enrichment tools (also GSEA). However, since radiation is a potent inducer of ROS, we still considered it relevant to pinpoint genes potentially related to ROS formation and a ROS response. The GO biological response gene set was therefore chosen as "a template" of oxidative stress due to its broad annotation of ROS-related genes (>400).

------------------------------------------------------------------------------------------------------------------------------------

**To reviewer #2:**

**Thank you for your comment and for taking time reviewing this manuscript.**

1. Authors mention in the Methods section that blood was harvested as well. What measurements were performed on these samples and if relevant why the data is not presented?

**Response to comment #1.**

Blood was collected for genotoxic assessments (i.a. Comet assay and flow cytometry-based micronucleus assay). These results will be presented in a separate manuscript already in preparation.

2. Is there any data in the literature concerning the in vitro radiation response of hepatocyte cell lines in terms of gene expression? If yes, authors should discuss it and compare it to their findings.

**Response to comment #2.**

Thank you for this comment. We have now performed a PubMed literature search to identify relevant in vitro radiation experiments using hepatocyte cell lines. After thorough considerations, we have concluded not to include more in vitro studies in the discussion.

Published studies using relevant cell lines exposed to radiation were identified. However, we did not discover studies utilizing relevant exposure regimes; low dose rate and low to moderate total dose, but mainly cancer-related studies using high dosing. Another vital aspect is the systemic responses after whole-body radiation, as used in our experiment. The hepatic gene expression profiles identified in this manuscript are probably connected to the holistic radiation-induced response, rather than a response to direct radiation in the liver alone, being a sensing organ for the whole body.

3. Where there any physiological parameters monitored during the course of the experiment? Weight, survival rate?

**Response to comment #3.**

The mice were monitored and cared for daily, and no mice died or showed clinical signs due to the exposure. Physiological parameters such as body weights were measured, and these results are presented in the manuscript focusing on the genotoxic effects.

------------------------------------------------------------------------------------------------------------------------------------
