# Supplementary material for: Perturbed transcriptional profiles after chronic low dose rate radiation in mice
Source: PLoS One. 2021 Aug 24;16(8):e0256667. doi: 10.1371/journal.pone.0256667 (PMC8384182; doi:10.1371/journal.pone.0256667)
Supplement: Peer review history

# Peer review: Decision Letter 0

**DOI:** 10.1371/journal.pone.0256667.r001

**Authors:** Tim A. Mousseau

5 Jul 2021

PONE-D-21-17536

Perturbed transcriptional profiles after chronic low dose rate radiation in mice

PLOS ONE

Dear Dr. Dahl,

Thank you for submitting your manuscript to PLOS ONE. After careful consideration, we feel that it has merit but does not fully meet PLOS ONE’s publication criteria as it currently stands. Therefore, we invite you to submit a revised version of the manuscript that addresses the points raised during the review process.

Based on the reviewer's comments, this paper is on good shape and falls between major and minor revisions. I believe almost all of the reviewers' suggestions can be addressed without requiring any new lab studies. However, suggestion #4 from reviewer #1 might require some additional work:

"4.) Confirmation of the RNA-sequencing via alternative non-high-throughput measurements (qRT-PCR, protein expression) would enhance these results."

Is this feasible? Perhaps this has already been done? Perhaps you don't feel this is neccessary?

Given the significance of this study, it would be very helpful to address this and the other issues as rigorously as possible.

Please submit your revised manuscript by Aug 19 2021 11:59PM. If you will need more time than this to complete your revisions, please reply to this message or contact the journal office at plosone@plos.org. When you're ready to submit your revision, log on to https://www.editorialmanager.com/pone/ and select the 'Submissions Needing Revision' folder to locate your manuscript file.

Please include the following items when submitting your revised manuscript:

- A rebuttal letter that responds to each point raised by the academic editor and reviewer(s). You should upload this letter as a separate file labeled 'Response to Reviewers'.
- A marked-up copy of your manuscript that highlights changes made to the original version. You should upload this as a separate file labeled 'Revised Manuscript with Track Changes'.
- An unmarked version of your revised paper without tracked changes. You should upload this as a separate file labeled 'Manuscript'.

If you would like to make changes to your financial disclosure, please include your updated statement in your cover letter. Guidelines for resubmitting your figure files are available below the reviewer comments at the end of this letter.

If applicable, we recommend that you deposit your laboratory protocols in protocols.io to enhance the reproducibility of your results. Protocols.io assigns your protocol its own identifier (DOI) so that it can be cited independently in the future. For instructions see: http://journals.plos.org/plosone/s/submission-guidelines#loc-laboratory-protocols. Additionally, PLOS ONE offers an option for publishing peer-reviewed Lab Protocol articles, which describe protocols hosted on protocols.io. Read more information on sharing protocols at https://plos.org/protocols?utm_medium=editorial-email&utm_source=authorletters&utm_campaign=protocols.

We look forward to receiving your revised manuscript.

Kind regards,

Tim A. Mousseau

Academic Editor

PLOS ONE

Journal Requirements:

When submitting your revision, we need you to address these additional requirements.

1. Please ensure that your manuscript meets PLOS ONE's style requirements, including those for file naming. The PLOS ONE style templates can be found at 

https://journals.plos.org/plosone/s/file?id=wjVg/PLOSOne_formatting_sample_main_body.pdf and 

https://journals.plos.org/plosone/s/file?id=ba62/PLOSOne_formatting_sample_title_authors_affiliations.pdf

2. Thank you for stating the following financial disclosure: 

"DO received fundings from the Research Council of Norway through its Centres of Excellence funding scheme, project number 223268/F50 CERAD. AKO received fundings from CERAD. 

AKO had a role in study design, decision to publish, and preparation of the manuscript. DO had a role in the preparation of the manuscript.

https://www.forskningsradet.no/en/.

.  " ext-link-type="uri" xlink:type="simple">https://www.nmbu.no/en/services/centers/cerad".

Please state what role the funders took in the study. If the funders had no role, please state: "The funders had no role in study design, data collection and analysis, decision to publish, or preparation of the manuscript." 

If this statement is not correct you must amend it as needed. 

Please include this amended Role of Funder statement in your cover letter; we will change the online submission form on your behalf.

3. We note that you have stated that you will provide repository information for your data at acceptance. Should your manuscript be accepted for publication, we will hold it until you provide the relevant accession numbers or DOIs necessary to access your data. If you wish to make changes to your Data Availability statement, please describe these changes in your cover letter and we will update your Data Availability statement to reflect the information you provide.

4. We note that you have included the phrase “data not shown” in your manuscript. Unfortunately, this does not meet our data sharing requirements. PLOS does not permit references to inaccessible data. We require that authors provide all relevant data within the paper, Supporting Information files, or in an acceptable, public repository. Please add a citation to support this phrase or upload the data that corresponds with these findings to a stable repository (such as Figshare or Dryad) and provide and URLs, DOIs, or accession numbers that may be used to access these data. Or, if the data are not a core part of the research being presented in your study, we ask that you remove the phrase that refers to these data.

[Note: HTML markup is below. Please do not edit.]

Reviewers' comments:

Reviewer's Responses to Questions

**Comments to the Author**

1. Is the manuscript technically sound, and do the data support the conclusions?

The manuscript must describe a technically sound piece of scientific research with data that supports the conclusions. Experiments must have been conducted rigorously, with appropriate controls, replication, and sample sizes. The conclusions must be drawn appropriately based on the data presented. 

Reviewer #1: Yes

Reviewer #2: Yes

**********

2. Has the statistical analysis been performed appropriately and rigorously? 

Reviewer #1: Yes

Reviewer #2: Yes

**********

3. Have the authors made all data underlying the findings in their manuscript fully available?

The PLOS Data policy requires authors to make all data underlying the findings described in their manuscript fully available without restriction, with rare exception (please refer to the Data Availability Statement in the manuscript PDF file). The data should be provided as part of the manuscript or its supporting information, or deposited to a public repository. For example, in addition to summary statistics, the data points behind means, medians and variance measures should be available. If there are restrictions on publicly sharing data—e.g. participant privacy or use of data from a third party—those must be specified.

Reviewer #1: Yes

Reviewer #2: Yes

**********

4. Is the manuscript presented in an intelligible fashion and written in standard English?

PLOS ONE does not copyedit accepted manuscripts, so the language in submitted articles must be clear, correct, and unambiguous. Any typographical or grammatical errors should be corrected at revision, so please note any specific errors here.

Reviewer #1: Yes

Reviewer #2: Yes

**********

5. Review Comments to the Author

Please use the space provided to explain your answers to the questions above. You may also include additional comments for the author, including concerns about dual publication, research ethics, or publication ethics. (Please upload your review as an attachment if it exceeds 20,000 characters)

Reviewer #1: The authors provide a novel hepatic transcriptional study with a chronic low dose rate in two mice strains at 24 hours and 100 days post-radiation exposure. The radiation endpoint was chosen to deliver the same total dose (3Gy) and provides an interesting direct comparison of dose-rate effects. The manuscript revealed a number of DEGs at 24 hours post-radiation exposure in both mice strains and discussed several functional pathways through enrichment analyses. However, at the second timepoint (100 days) there were few DEGs and interpretation of functionally relevant pathways was limited. This may be expected given the long recovery time post-exposure, but is also confounded by the large time range when the mice were terminated (106-221 days). There was a brief comment about “breeding inter- and trans-generational study groups”(line 142) in the methods section that may have influenced this result. While the authors also measured and found no statically significant changes in global DNA methylation, the results of this study are solely based on bioinformatic and statistical analysis of DEGs from RNA-sequencing. The strain-related differences/similarities in genes and pathways that were statistically significant add to the previously ascribed radiation resistance of the C57BL/6 strain found at higher doses and dose-rates.

Major Comments:

1.) It would be useful to display the DEGs for each condition as a volcano plot to quickly visualize the fold-changes in relationship to the significance rather than referring to the supplemental data table. A hierarchical clustered heat map for the total DEGs per mouse strain (2,608 for CBA and 1,449 for B6) may also provide a useful alternative.

2.) The authors state “no log2FC cut-off value was used” (line 198) when determining the direction of expression for the gene expression. However, based on the supplemental data provided, there was an inherent cut-off perhaps due to using an FDR 0.5.

3.) Are there any connections to the DEGs uncovered in this study and the related study on miRNAs perturbed by chronic low-dose irradiation (Duale et al. 2020)? While that study was examining biomarkers of LDR irradiation, any connections might strengthen the results found in the transcriptional study.

4.) Confirmation of the RNA-sequencing via alternative non-high-throughput measurements (qRT-PCR, protein expression) would enhance these results.

5.) A limitation of pathway enrichment analysis is the dependence on predefined gene sets which led the authors to focus on the Biological Process GO Term “Response to oxidative stress” (GO:0006979) rather than relying on the KEGG database which lacks an ROS category. An alternative may include analyzing the current dataset with the Molecular Signatures Database (MSigDB) and performing a Gene Set Enrichment Analysis (Subramanian et al., Gene set enrichment analysis: A knowledge-based approach for interpreting genome-wide expression profiles, PNAS, 2005). This database includes many genesets other than KEGG and GO that may support the current analysis.

Reviewer #2: 1. Authors mention in the Methods section that blood was harvested as well. What measurements were performed on these samples and if relevant why the data is not presented?

2. Is there any data in the literature concerning the in vitro radiation response of hepatocyte cell lines in terms of gene expression ? If yes, authors should discuss it and compare it to their findings.

3. Where there any physiological parameters monitored during the course of the experiment? Weight, survival rate?

**********

6. PLOS authors have the option to publish the peer review history of their article (what does this mean?). If published, this will include your full peer review and any attached files.

If you choose “no”, your identity will remain anonymous but your review may still be made public.

**Do you want your identity to be public for this peer review?** For information about this choice, including consent withdrawal, please see our Privacy Policy.

Reviewer #1: No

Reviewer #2: No

[NOTE: If reviewer comments were submitted as an attachment file, they will be attached to this email and accessible via the submission site. Please log into your account, locate the manuscript record, and check for the action link "View Attachments". If this link does not appear, there are no attachment files.]

While revising your submission, please upload your figure files to the Preflight Analysis and Conversion Engine (PACE) digital diagnostic tool, https://pacev2.apexcovantage.com/. PACE helps ensure that figures meet PLOS requirements. To use PACE, you must first register as a user. Registration is free. Then, login and navigate to the UPLOAD tab, where you will find detailed instructions on how to use the tool. If you encounter any issues or have any questions when using PACE, please email PLOS at figures@plos.org. Please note that Supporting Information files do not need this step.

# Author comment: Author response to Decision Letter 0

**DOI:** 10.1371/journal.pone.0256667.r002

10 Aug 2021

Response to editor and reviewers

Comments to reviewers:

Please note that the revised manuscript has changed page, line, and figure numbering and is no longer following the previous indications. In addition to the revisions based on the reviewers’ and requests, minor text editing has been made throughout the manuscript to ensure clarity and accurate grammar. All changes are tracked, and the line referencing is related to the manuscript with tracked changes. 

Response to editor’s comment:

"...suggestion #4 from reviewer #1 might require some additional work: "4.) Confirmation of the RNA-sequencing via alternative non-high-throughput measurements (qRT-PCR, protein expression) would enhance these results." Is this feasible? Perhaps this has already been done? Perhaps you don't feel this is necessary?”

Thank you for this comment. We have now added results from a qPCR validation analysis of a selection of genes to the manuscript. For further details, see comment #4, reviewer #1.

------------------------------------------------------------------------------------------------------------------------------------

To reviewer #1:

Thank you for your comments and for taking time reviewing this manuscript.

Reviewer #1: The authors provide a novel hepatic transcriptional study with a chronic low dose rate in two mice strains at 24 hours and 100 days post-radiation exposure. The radiation endpoint was chosen to deliver the same total dose (3Gy) and provides an interesting direct comparison of dose-rate effects. The manuscript revealed a number of DEGs at 24 hours post-radiation exposure in both mice strains and discussed several functional pathways through enrichment analyses. However, at the second timepoint (100 days) there were few DEGs and interpretation of functionally relevant pathways was limited. This may be expected given the long recovery time post-exposure, but is also confounded by the large time range when the mice were terminated (106-221 days). There was a brief comment about “breeding inter- and trans-generational study groups ”(line 142) in the methods section that may have influenced this result. While the authors also measured and found no statically significant changes in global DNA methylation, the results of this study are solely based on bioinformatic and statistical analysis of DEGs from RNA-sequencing. The strain-related differences/similarities in genes and pathways that were statistically significant add to the previously ascribed radiation resistance of the C57BL/6 strain found at higher doses and dose-rates.

1.) It would be useful to display the DEGs for each condition as a volcano plot to quickly visualize the fold-changes in relationship to the significance rather than referring to the supplemental data table. A hierarchical clustered heat map for the total DEGs per mouse strain (2,608 for CBA and 1,449 for B6) may also provide a useful alternative. 

Response to comment #1.

Thank you for your suggestion for better visualization of the data. A volcano plot for each condition one day post-radiation has been added to the manuscript; named Fig 3, the caption is found in lines 277 – 280, page no: 12. All the following figure numbering are accordingly changed. 

2.) The authors state “no log2FC cut-off value was used” (line 198) when determining the direction of expression for the gene expression. However, based on the supplemental data provided, there was an inherent cut-off perhaps due to using an FDR 0.5.

Response to comment #2.

Thank you for noticing the unclear description in the section for statistical analyses. The section for “statistical analysis” has now been clarified as modifications are made to the text: lines 218 – 249, pages no: 9 – 11. 

3.) Are there any connections to the DEGs uncovered in this study and the related study on miRNAs perturbed by chronic low-dose irradiation (Duale et al. 2020)? While that study was examining biomarkers of LDR irradiation, any connections might strengthen the results found in the transcriptional study.

Response to comment #3. 

Thank you for this suggestion. We have included a correlation analysis in determining an interaction between the predicted miRNA in Duale et al. 2020 and our identified genes. A figure has been added as supplementary: S2 Figure. The correlation analysis is mentioned in the result section, page no. 12, lines 304 - 311.

4.) Confirmation of the RNA-sequencing via alternative non-high-throughput measurements (qRT-PCR, protein expression) would enhance these results.

Response to comment #4.

We have now performed validation of a selection of DEGs using qPCR. A correlation Scatter plot has been added to the manuscript as Supplementary: S3 Figure. 

The qPCR data are in high concordance with the RNA-Seq data. Hence, we consider that the RNA-Seq data performed at high sequencing depth (85 million reads per sample) gives reliable results, rendering a full qPCR confirmation redundant. After thorough considerations, we concluded that verification of all the statistically significant DEGs is unnecessary.

As these modifications require adding additional information to the section “material and method”, the subsection "Quantitative real-time PCR" has been added to page no. 8, line 187 to page 9, line 207. 

The validation is reported in the result section, page no. 12, lines 312 - 313. 

5.) A limitation of pathway enrichment analysis is the dependence on predefined gene sets which led the authors to focus on the Biological Process GO Term “Response to oxidative stress” (GO:0006979) rather than relying on the KEGG database which lacks an ROS category. An alternative may include analyzing the current dataset with the Molecular Signatures Database (MSigDB) and performing a Gene Set Enrichment Analysis (Subramanian et al., Gene set enrichment analysis: A knowledge-based approach for interpreting genome-wide expression profiles, PNAS, 2005). This database includes many genesets other than KEGG and GO that may support the current analysis.

Response to comment #5. 

Thank you for this comment. The sub-section of “Oxidative stress” under “Results” has now been clarified as modifications are made to the text: page 18, line 412 - 421.

In the process of analyzing and evaluating our RNASeq data, a range of different bioinformatic strategies and enrichment tools were tested and evaluated (over-representation (ORA), ranking, and topological), including GSEA. EnrichR, as GSEA, includes a wide range of gene set libraries (also MSigDB). However, the statistical calculations are more simplified than GSEA, as EnrichR uses hypergeometric testing. 

However, predefined gene sets related to a ROS category were not significant when evaluating output from different enrichment tools (also GSEA). However, since radiation is a potent inducer of ROS, we still considered it relevant to pinpoint genes potentially related to ROS formation and a ROS response. The GO biological response gene set was therefore chosen as "a template" of oxidative stress due to its broad annotation of ROS-related genes (400). 

------------------------------------------------------------------------------------------------------------------------------------

To reviewer #2: 

Thank you for your comment and for taking time reviewing this manuscript.

1. Authors mention in the Methods section that blood was harvested as well. What measurements were performed on these samples and if relevant why the data is not presented?

Response to comment #1. 

Blood was collected for genotoxic assessments (i.a. Comet assay and flow cytometry-based micronucleus assay). These results will be presented in a separate manuscript already in preparation.

2. Is there any data in the literature concerning the in vitro radiation response of hepatocyte cell lines in terms of gene expression? If yes, authors should discuss it and compare it to their findings. 

Response to comment #2. 

Thank you for this comment. We have now performed a PubMed literature search to identify relevant in vitro radiation experiments using hepatocyte cell lines. After thorough considerations, we have concluded not to include more in vitro studies in the discussion.

Published studies using relevant cell lines exposed to radiation were identified. However, we did not discover studies utilizing relevant exposure regimes; low dose rate and low to moderate total dose, but mainly cancer-related studies using high dosing. Another vital aspect is the systemic responses after whole-body radiation, as used in our experiment. The hepatic gene expression profiles identified in this manuscript are probably connected to the holistic radiation-induced response, rather than a response to direct radiation in the liver alone, being a sensing organ for the whole body.

3. Where there any physiological parameters monitored during the course of the experiment? Weight, survival rate?

Response to comment #3. 

The mice were monitored and cared for daily, and no mice died or showed clinical signs due to the exposure. Physiological parameters such as body weights were measured, and these results are presented in the manuscript focusing on the genotoxic effects.

------------------------------------------------------------------------------------------------------------------------------------

Attachment Submitted filename: Response_to_Reviewers.docx Click here for additional data file.

# Editor report: Decision Letter 1

**DOI:** 10.1371/journal.pone.0256667.r003

**Authors:** Tim A. Mousseau

12 Aug 2021

Perturbed transcriptional profiles after chronic low dose rate radiation in mice

PONE-D-21-17536R1

Dear Dr. Dahl,

We’re pleased to inform you that your manuscript has been judged scientifically suitable for publication and will be formally accepted for publication once it meets all outstanding technical requirements. Nice work, and congratulations!

Within one week, you’ll receive an e-mail detailing the required amendments. When these have been addressed, you’ll receive a formal acceptance letter and your manuscript will be scheduled for publication.

An invoice for payment will follow shortly after the formal acceptance. To ensure an efficient process, please log into Editorial Manager at http://www.editorialmanager.com/pone/, click the 'Update My Information' link at the top of the page, and double check that your user information is up-to-date. If you have any billing related questions, please contact our Author Billing department directly at authorbilling@plos.org.

If your institution or institutions have a press office, please notify them about your upcoming paper to help maximize its impact. If they’ll be preparing press materials, please inform our press team as soon as possible -- no later than 48 hours after receiving the formal acceptance. Your manuscript will remain under strict press embargo until 2 pm Eastern Time on the date of publication. For more information, please contact onepress@plos.org.

Kind regards,

Tim A. Mousseau

Academic Editor

PLOS ONE

# Editor report: Acceptance letter

**DOI:** 10.1371/journal.pone.0256667.r004

**Authors:** Tim A. Mousseau

16 Aug 2021

PONE-D-21-17536R1 

Perturbed transcriptional profiles after chronic low dose rate radiation in mice 

Dear Dr. Dahl:

I'm pleased to inform you that your manuscript has been deemed suitable for publication in PLOS ONE. Congratulations! Your manuscript is now with our production department. 

If your institution or institutions have a press office, please let them know about your upcoming paper now to help maximize its impact. If they'll be preparing press materials, please inform our press team within the next 48 hours. Your manuscript will remain under strict press embargo until 2 pm Eastern Time on the date of publication. For more information please contact onepress@plos.org.

If we can help with anything else, please email us at plosone@plos.org. 

Thank you for submitting your work to PLOS ONE and supporting open access. 

Kind regards, 

PLOS ONE Editorial Office Staff

on behalf of

Dr. Tim A. Mousseau 

Academic Editor

PLOS ONE
